# Engineered nanoparticles promote cardiac tropism of AAV vectors

**DOI:** 10.1186/s12951-024-02485-6

**Published:** 2024-05-03

**Authors:** Lauren Switala, Lin Di, Huiyun Gao, Courteney Asase, Matthew Klos, Palanivel Rengasamy, Daria Fedyukina, Andrei Maiseyeu

**Affiliations:** 1 Department of Medicine, School of Medicine, Cardiovascular Research Institute, Case Western Reserve University, Cleveland, USA; 2 Department of Biomedical Engineering, Case Western Reserve University, Cleveland, USA; 3 Department of Pediatrics, Case Western Reserve University, Cleveland, USA; 4 Bioheights LLC, Cleveland, USA; 5 Present Address: Advanced Research Projects Agency for Health, ARPA-H, Washington, USA

## Abstract

Cardiac muscle targeting is a notoriously difficult task. Although various nanoparticle (NP) and adeno-associated viral (AAV) strategies with heart tissue tropism have been developed, their performance remains suboptimal. Significant off-target accumulation of i.v.-delivered pharmacotherapies has thwarted development of disease-modifying cardiac treatments, such as gene transfer and gene editing, that may address both rare and highly prevalent cardiomyopathies and their complications. Here, we present an intriguing discovery: cargo-less, safe poly (lactic-co-glycolic acid) particles that drastically improve heart delivery of AAVs and NPs. Our lead formulation is referred to as ePL (enhancer polymer). We show that ePL increases selectivity of AAVs and virus-like NPs (VLNPs) to the heart and de-targets them from the liver. Serotypes known to have high (AAVrh.74) and low (AAV1) heart tissue tropisms were tested with and without ePL. We demonstrate up to an order of magnitude increase in heart-to-liver accumulation ratios in ePL-injected mice. We also show that ePL exhibits AAV/NP-independent mechanisms of action, increasing glucose uptake in the heart, increasing cardiac protein glycosylation, reducing AAV neutralizing antibodies, and delaying blood clearance of AAV/NPs. Current approaches utilizing AAVs or NPs are fraught with challenges related to the low transduction of cardiomyocytes and life-threatening immune responses; our study introduces an exciting possibility to direct these modalities to the heart at reduced i.v. doses and, thus, has an unprecedented impact on drug delivery and gene therapy. Based on our current data, the ePL system is potentially compatible with any therapeutic modality, opening a possibility of cardiac targeting with numerous pharmacological approaches.

**Graphical Abstract:**

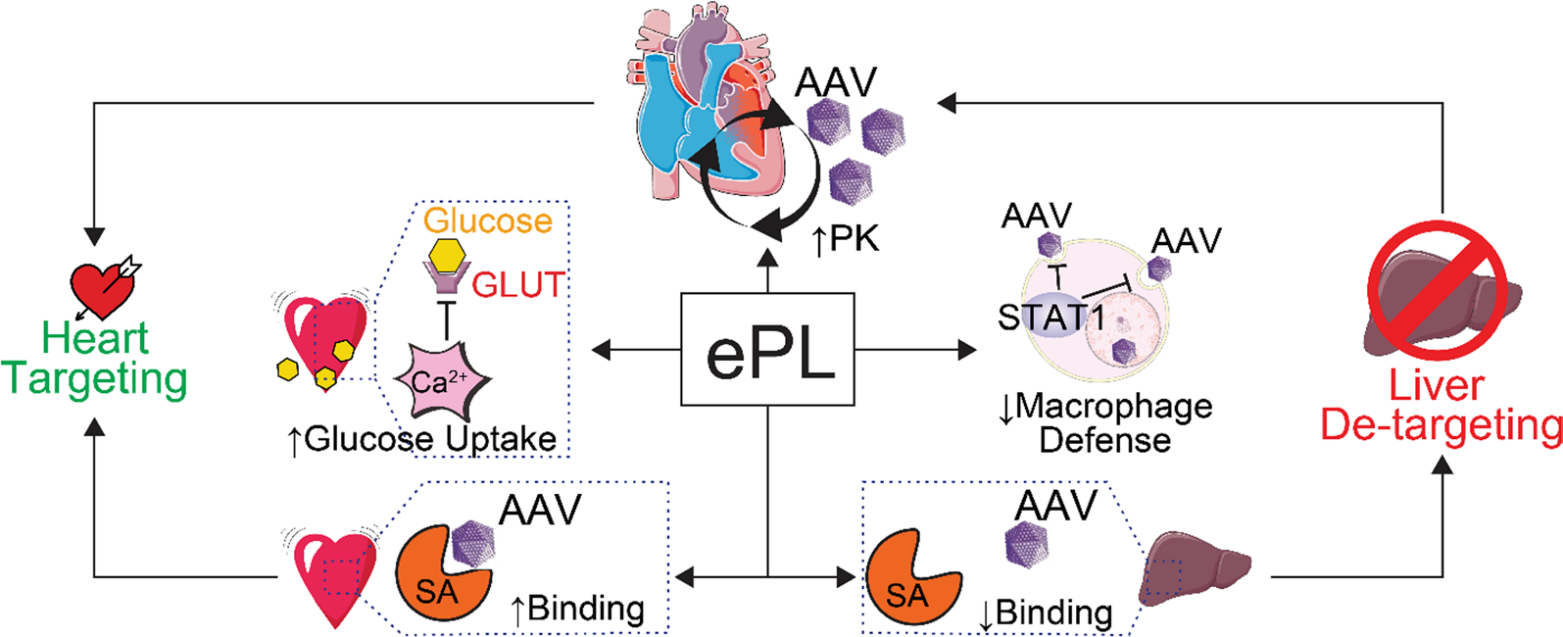

**Supplementary Information:**

The online version contains supplementary material available at 10.1186/s12951-024-02485-6.

## Introduction

Many promising therapeutic modalities for cardiomyopathies require targeted delivery of pharmacotherapy to the heart. As evidenced from the review of clinical trials, such therapeutic modalities have been administered either directly to the heart or systemically. Intracoronary and intramyocardial injections are highly invasive and not feasible for treatments that require frequent administrations. These invasive routes of administration are often not conducive to performing placebo-controlled studies and have been shown to discourage patient enrollment. On the other hand, systemically (intravenously, i.v.) administered treatments lead to a wide biodistribution, high off-target deposition in reticuloendothelial organs (RES), low therapeutic index, and undesirable side effects. Often, the i.v. route of administration necessitates higher and more frequent dosing. For one-and-done treatments such as adeno-associated viral (AAV) gene therapies or nanoparticles carrying gene editing machinery, improving heart targeting becomes critical to achieve adequate efficacy with a single dose. Numerous reports on intravenously injected AAV9, which has one of the best-known cardiac tropisms, have shown low heart-to-liver delivery ratios [1] requiring high viral doses. Such dose levels elicit life-threatening immune responses and liver pathology [2]. In general, enhancing heart-targeting and minimizing off-targeting may significantly improve pharmacotherapies for cardiac diseases.

To date, two main pre-clinical approaches to increase the amount of drug reaching the heart include optimization of a drug’s affinity to the heart [3–7] and reduction of off-targeting by detargeting the liver [8, 9]. To address the former, a number of research groups are searching for better targets that are highly overexpressed in diseased hearts [7]. Major efforts are ongoing to enhance cardiac muscle tropism of AAV vectors [3–5] and to engineer targeted iPSC-derived cardiomyocytes [6]. AAV capsid engineering, especially direct evolution engineering, can generate highly specific serotypes that target muscle and/or heart [3]. However, new capsid engineering is a laborious process of screening millions of capsid variants before the desired specificity is met [3]. This is a multi-species process because once optimal capsids are identified in mice, their translation to nonhuman primates and humans is not guaranteed [3]. For nanoparticle (NP) targeting, typical approaches include modification of the NP surface with an affinity ligand (antibody, peptide) that is designed to recognize targets in the heart [10–12]. Even though tissue specific targeting has been improved with active targeting strategies such as ligand-receptor recognition, it is often challenging to identify corresponding receptors on targeted cell types [13]. Often, antigenic ligands are designed for testing in mice on murine targets, which creates additional hurdles for potential translation in human patients [14]. Even with tissue-specific active targeting strategies, liver off-target deposition (i.e., the first-pass effect) remains the bottleneck for efficacious and safe delivery [15, 16].

One of the most attractive strategies for heart targeting that does not depend on antigenic recognition and is conserved between species is targeting through the glycosylation-GLUT axis. Therapeutic modalities that express carbohydrate exteriors, including glucose, [15] are able to target the GLUT transporters (e.g., GLUT1 and GLUT4) that are highly expressed in the heart. Unfortunately, targeting of GLUT transporters have been explored primarily in the context of tumor targeting due to tumor preference to glucose metabolism (i.e., upregulated Warburg effect.) [17] The general strategy for heart targeting with glucose as a ligand has been recently validated in experimental animals for ischemic heart disease treated with poly-glucose nanoparticles. [18]

Another important consideration is glycosylation of the tissue itself, i.e., the expression of glycoconjugates on cell surfaces, which has been shown to be important for AAV tropism. It is well known that many AAV serotypes utilize glycoconjugates, including sialic acid, as receptors [19].

To address the issues with off-targeting, various strategies have been developed that reduce phagocytic clearance by Kupffer cells (KCs) and liver sinusoidal endothelial cells (LSECs). Unfortunately, there are only a few examples of non-AAV approaches that explored active liver detargeting applied to an i.v.-administered therapy. In one example, to improve gene therapy targeting, Robert Langer’s group used a “nano primer” which is administered prior to lipid nanoparticles (LNPs) carrying mRNA. The nanoprimer is taken up by KCs and LSECs, which allows the LNPs to reach and transduce the hepatocytes [20]. The mechanism of action is described as “*blocking the RES in the liver*” [20], which denotes the impairment of clearance mechanisms due to liver cells being “overwhelmed” by the high dose of the nano primer. A somewhat similar approach is described by Stephan Grabbe and colleagues, who injected an FcR-blocking agent prior to injecting NPs, resulting in a significant liver NP detargeting [21]. Both approaches take advantage of an intrinsic ability of phagocytic liver cells to take up a bulk of non-therapeutic (sham) NPs to prevent the uptake of therapeutic cargo-carrying NPs. Remarkably, detargeting and targeting were accomplished in the same organ (i.e., the liver): the aforementioned liver detargeting technologies were developed to enable specific targeting of hepatocytes. This “bait and switch” concept is a viable method to decrease off-targeting and increase on-targeting, and would be especially valuable for targeting of a non-liver organ, such as the heart.

Herein, we describe a novel strategy that leverages the aforementioned glycosylation-GLUT axis for targeting and multiple mechanisms of liver detargeting. We accomplish this with an engineered enhancer polymer (ePL) that circumvents major hurdles of conventional drug delivery systems that rely on active targeting (e.g., via epitope recognition) or serotype engineering for AAV. Our strategy comprises two sequentially administered injectable components: (1) ePL—precision-engineered non-racemic poly (lactic-co-glycolic acid) particles (PLGA) and (2) a glycosylated therapeutic modality, such as a small molecule, a nanoparticle (NP), or an AAV vector. ePL produces heart targeting and liver detargeting effects without the need to modify AAVs (other therapeutic moieties are minimally modified with glycosylation), thus creating opportunities to enhance gene therapies that have been previously validated for efficacy but thwarted by poor safety or high production costs. ePL has a potential to enhance a wide variety of AAV serotypes, and thus represents a complementary approach to novel serotype discovery efforts.

## Results

### Model fluorescent nanoparticles target the heart if co-administered with PLGA

**Fig. 1:**
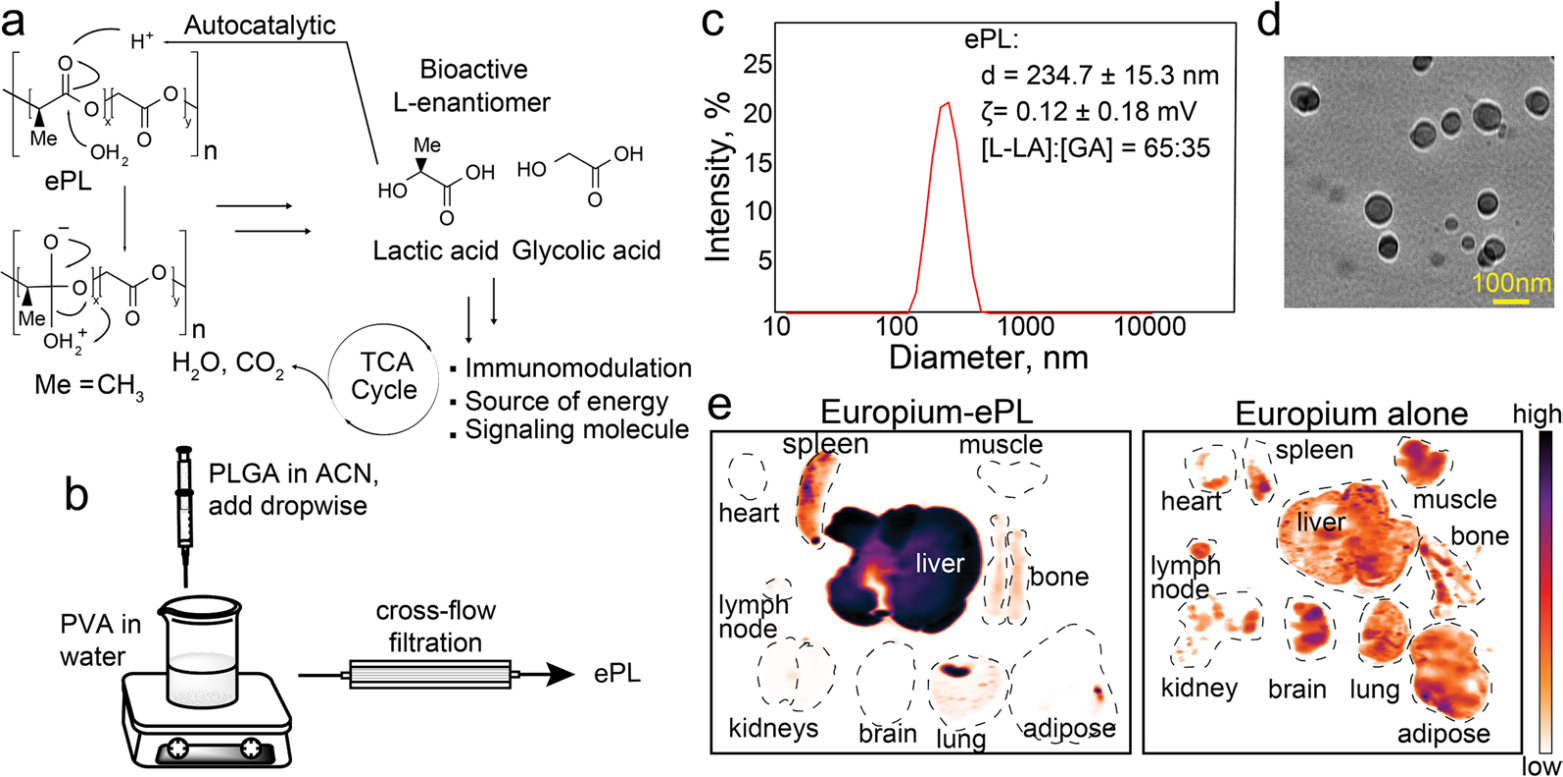
ePL synthesis and characterization. **a** Schematic showing PLGA composition and degradation in vivo. **b** Schematic of ePL particle synthesis by nanoprecipitation. **c** Size distribution and zeta potential of ePL by DLS. **d** Transmission electron microscopy (TEM) examination of ePL shows that ePL particles are spherical in shape. **e** Biodistribution of ePL labeled with fluorescent europium cryptate 1 h after i.v. injection, showing significant accumulation in the liver (n = 3 animals per group)

PLGA polymers are attractive drug delivery vehicles, especially in the form of nanoparticles, because of their excellent safety profile and ability to spontaneously degrade in biological systems into lactic acid and glycolic acid (Fig. 1a) [22]. We were interested in utilizing PLGA nanoparticles not as drug delivery vehicles but as a cargo-less aid to existing therapies. A previous study showed co-delivery of polymer-based “nanoprimers” lead to reduced clearance of therapeutic LNPs. [20] In a proof-of-concept experiment, we first investigated biodistribution of co-delivered various non-PLGA nanoparticles and nanoparticles made of PLGA. We observed an intriguing heart-biased biodistribution pattern of model polystyrene latex fluorospheres that were co-injected with PLGA nanoparticles. To gain insight into this phenomenon, we synthesized different cargo-less PLGA nanoparticle formulations of varying polymer molecular weight. We co-injected these particles with 25 nm fluorescent latex beads into C57BL/6 mice i.v.. Hearts were excised 1 h post injection (p.i.) and fluorescence was recorded using whole organ imaging following heart perfusion (Additional file 1: Fig. S1a). Larger PLGA polymers (120 kDa) enhanced the uptake of the latex beads in the heart (Additional file 1: Fig. S1b,c). Based on these initial findings, 120 kDa poly(L-lactic-co-glycolic acid) (PLLGA) with a lactic to glycolic ratio of 65:35 was chosen for future experiments. For simplicity, we refer to the nanoparticles made from this polymer as ePL (enhancer polymer). ePL was synthesized by means of standard flash nanoprecipitation from acetonitrile to polyvinyl alcohol (PVA) surfactant in water (Fig. 1b). ePL was characterized by dynamic light scattering (DLS) to measure the particle size and zeta potential (Fig. 1c). The diameter of the particles was determined to be 234.7 ± 15.3 nm, while the zeta potential was 0.12 ± 0.18 mV. Using transmission electron microscopy (TEM), we determined that the particles had a spherical shape (Fig. 1d). Next, we studied the biodistribution of ePL using a fluorescently-labeled ePL. We conjugated PLGA to a europium (Eu) fluorescent dye and blended 5% of Eu-labeled PLGA with unlabeled ePL. After the injection at 10 mg/kg in C57BL/6 mice, followed by perfusion, organ excision, and imaging (Fig. 1e), we found that ~ 90% of ePL accumulated in the liver and ~ 5% in the spleen. No ePL deposition in the heart, brain, or muscle was observed. The clearance of ePL from the systemic circulation was very rapid (T_1/2_ = 5.3 ± 2.6 min), suggesting that ePL has a high liver affinity. We did not expect significant toxicities associated with ePL because it is based on poly-lactide, which is highly biodegradable and considered safe [22, 23]. Examination of histological staining of heart and liver sections from mice bolus-injected with 30 mg/kg ePL revealed that these tissues appeared all normal following H&E staining, with no obvious sign of fibrosis or cell infiltrations (Additional file 1: Fig. S1d). Because ePL is highly biodegradable, a hepatobiliary route of elimination was observed (Additional file 1: Fig. S1e). Finally, because of significant ePL accumulation in the spleen (Fig. 1e), we tested for the possibility of splenic injury, immune cell infiltration and a splenic infarct. However, we noticed none of these effects on spleen gross pathology (Additional file 1: Fig. S1f) even at high concentrations of ePL. ePL was free from endotoxin contamination (Additional file 1: Fig. S1g).

### ePL enhances the heart tropism of AAV and virus-like nanoparticles

**Fig. 2:**
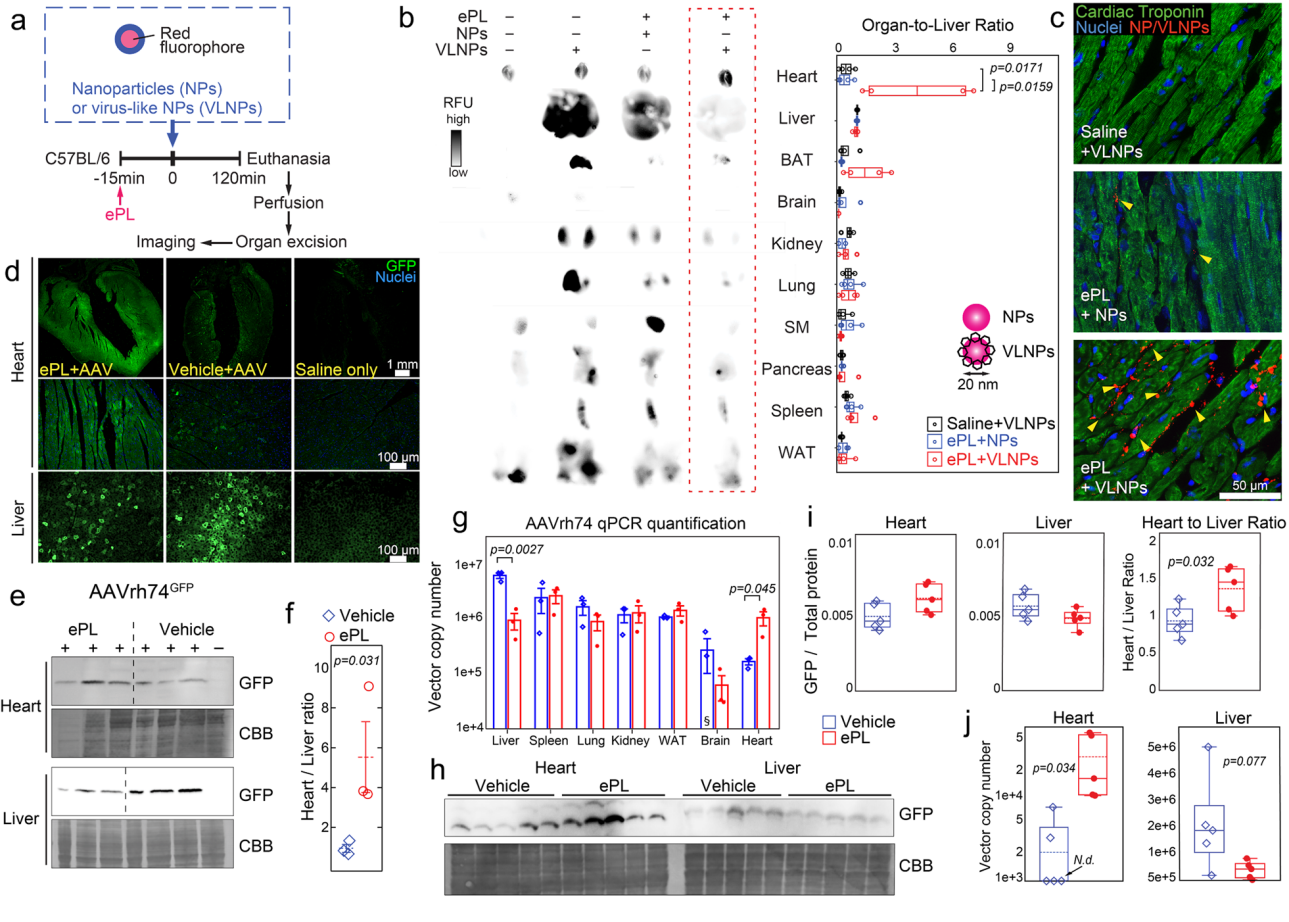
ePL facilitates heart targeting of virus-like nanoparticles (VLNP), AAVrh.74, and AAV1. **a** Optimized injection schedule to test VLNP distribution with ePL. **b** Biodistribution imaging in various organs of fluorescently-labeled VLNP 2 h after i.v. administration. Fluorescent intensity is quantified and normalized to organ fluorescence from non-injected animals. **c** Fluorescence microscopy of heart sections from mice injected as indicated. The sections were stained with anti-cardiac troponin (CT3) antibodies (green) co-registered with endogenous NP fluorescence (red, arrow-heads). Nuclei were visualized after staining with DAPI (blue). *BAT* brown adipose tissue, *WAT* white adipose tissue. n = 5–6 mice/group. **d** Immunofluorescence analysis of GFP protein expression in hearts and livers of ePL- or vehicle-injected mice 30 days after injection of a single dose of 5e11 vg/kg AAVrh74.CMV-eGFP. GFP expression was visualized and quantified after the staining with anti-GFP antibodies. **e** Western blot analysis of GFP protein in heart and liver lysates of AAVrh74.CMV-eGFP-injected animals. *CBB*  Coomassie brilliant blue. **f** Densitometry quantification of blots in e. **g** qPCR analysis of vector copy numbers (VCN) in various organs from AAVrh74-injected animals with and without ePL. § data point below range of plot (8.37E3 VCN) **h** Western blot analysis of GFP protein in heart and liver lysates of 5e11 vg/kg AAV1.CMV-GFP-injected animals. **i** Densitometry quantification of (**h**) from AAV1-injected animal heart and liver lysates, as well as the ratio of those organs. **j** qPCR analysis in animals injected with 5e11 vg/kg AAV1.CMV-eGFP. n = 3–6 mice/group

To determine whether the ePL heart-redirecting effect may impact both nanoparticle and AAV delivery, we used latex fluorospheres incorporating far red fluorophore (NPs) and virus-like nanoparticles (VLNPs), which we engineered and synthesized with glycosylated surface that mimicked an AAV capsid. Both NPs and VLNPs had an average diameter of ~ 25 nm, thus closely resembling the average size of AAV capsids. Upon optimization of the injection schedule (Fig. 2a), we discovered that administration of ePL 15 min before VLNPs significantly enhanced VLNP deposition in the heart and detargeted the liver (Fig. 2b, Additional file 1: Figure S2a). Other organs of the reticuloendothelial system (RES) were also detargeted. The effect was particularly striking when glycosylated VLNPs were used (Fig. 2b). Specifically, an injection of ePL before the injection of VLNP resulted in an order of magnitude increased accumulation of VLNP in the heart vs. the liver as evidenced by heart-to-liver ratio 0.41 ± 0.20 for VLNP vs. 4.16 ± 1.55 for ePL + VLNP (p = 0.017) (Fig. 2b). The VLNPs were confirmed to accumulate within cardiomyocytes using immunofluorescence in heart sections (Fig. 2c). Collectively, these data demonstrate that ePL is able to robustly redirect nanoparticles to the heart and detarget the liver and that this effect was dependent on surface glycosylation engineered for VLNPs.

Encouraged by the preliminary results on ePL-enhanced VLNP delivery (Fig. 2b, c) and safety (Additional file 1: Fig. S1), we set out to test the effect of ePL on AAV. In our pilot studies, we used the AAVrh.74 serotype injected i.v. because it has been clinically validated and exhibits cardiac muscle tropism. [24]

First, we tested the hypothesis that ePL would further increase the AAVrh.74 affinity to the heart. A single i.v. injection of 5e11 vg/kg (vector genomes per kilogram) single-stranded AAVrh74 carrying the eGFP transgene under the control of the CMV promoter (AAVrh74.CMV-eGFP, referred to as AAVrh.74 further in the text for simplicity) was administered following the injection schedule shown in Fig. 2a. Because the expression of the AAV-carrying transgene (eGFP in this case) becomes appreciable at four-to-six weeks following the delivery [1, 25], the eGFP protein expression was examined in the heart and the liver 30 days after the ePL and AAV injections. A 5.1 ± 0.3 fold increase (p < 0.001) in the eGFP expression in the heart was observed with ePL + AAVrh.74 as compared to animals that received AAVrh.74 alone (Fig. 2d, quantified in Additional file 1: Fig. S2b, c), as demonstrated by immunofluorescence studies using antibodies against the eGFP transgene. This targeting pattern was additionally confirmed by quantitative Western blotting using an antibody against eGFP and total protein staining (Fig. 2e, f) which demonstrated heart-to-liver ratios of 5.5 ± 1.0 for ePL + AAVrh.74 group vs. 0.9 ± 0.1 1.2 for AAVrh.74 alone (p = 0.031) (Fig. 2f). Further biodistribution studies 2 h p.i. were conducted by qPCR in major organs to detect AAVrh.74 DNA using primers and probes specific for CMV (Fig. 2g). The data demonstrated robust heart targeting and liver de-targeting with ePL and is suggestive of redirecting AAVrh.74 capsids from the liver to the heart, rather than acting on the eGFP expression in the heart. In these experiments, AAVrh.74 displayed a 6.5 ± 1.9-fold increase (p = 0.045) in heart targeting and a 6.8 ± 0.05-fold decrease in liver uptake (p = 0.0027) with ePL as compared to vehicle control (Fig. 2g). Interestingly, the data also demonstrated a trend towards lung detargeting. Finally, skeletal muscle uptake of AAVrh.74 was examined separately by Southern blotting (Additional file 1: Fig. S2d) due to the levels of viral DNA in the muscle being too low for traditional qPCR detection. The data demonstrated a significant 2.5 ± 0.8-fold increase (p = 0.041) in AAVrh.74 accumulation in the skeletal muscle with ePL as compared to the vehicle injection.

Next, we wanted to assess whether ePL-driven heart targeting is still possible with an AAV serotype known for its poor heart tropism in i.v.-injected mice. We selected AAV1 for this purpose [1, 26]. We administered AAV1.CMV.eGFP (AAV1) with and without ePL in the same manner as above, analyzing the protein expression 30 days p.i. (Fig. 2h, i) and AAV1 DNA levels 2 h p.i. in the heart and liver (Fig. 2j). Notably, on the eGFP protein expression level, ePL significantly improved the heart-to-liver ratio was 1.31 ± 0.14 for the ePL + AAV1 group vs. 0.89 ± 0.10 for the AAV1-only group (p = 0.032) (Fig. 2i). Strikingly, a 14.1 ± 0.4-fold increase (p = 0.034) in heart delivery with ePL (as compared to the AAV1-only control) was observed on the DNA level (Fig. 2j). In contrast, 3 of 5 injected animals in the AAV1-only group did not show any viral DNA in the heart, whereas high liver accumulation was detected in the same animals. Collectively, this data suggests that ePL pre-injection enables significant enhancement of AAV accumulation in the cardiac muscle for AAV serotypes with or without inherent cardiac tropism. We hypothesized that this heart targeting mechanism likely relies on the systemic distribution of ePL and AAV. This is suggested by the fact that we have not observed any evidence of ePL accumulation in the heart (Fig. 2b), and direct interactions of ePL with the viral particles are also unlikely due to the time delay between the ePL and AAV injections.

### ePL accumulates in Kupffer cells and produces serum-derived factors enhancing AAV uptake in cardiac/muscle cells

**Fig. 3:**
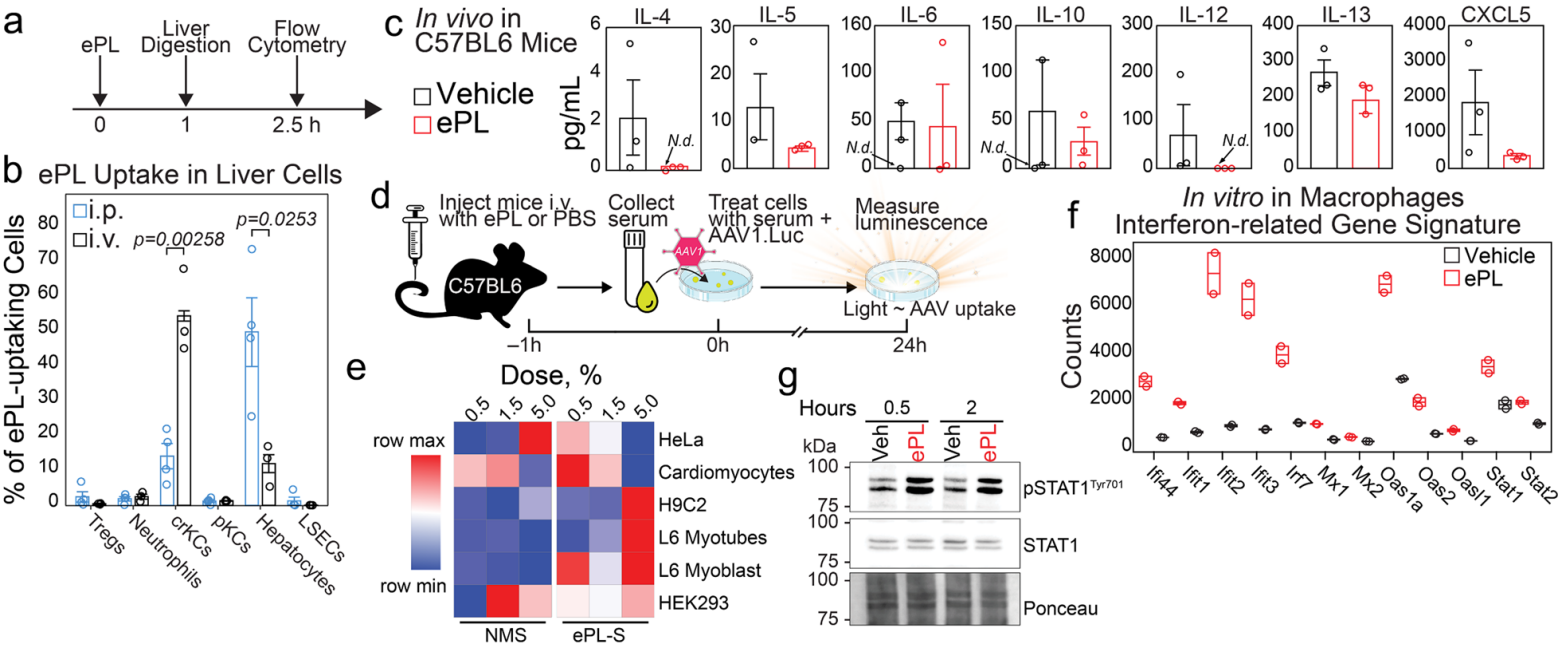
Intravenous administration of ePL targets cytokine-releasing KCs in the liver. **a** Mice were administered i.v. or intraperitoneally (i.p.) with fluorescently-labeled ePL at 70 mg/kg (n = 3 per group). **b** Livers were digested to obtain single cell suspension which was then analyzed using flow cytometry. Cell markers: regulatory T cells (Tregs): CD3^+^CD4^+^CD25^+^; Neutrophils: CD11b^+^F4/80^−^Ly6G^+^; Cytokine-releasing Kupffer Cells (crKCs): CD11b^+^F4/80^+^; Phagocytic Kupffer Cells (pKCs): CD11b^+^F4/80^+^CD68^+^; Hepatocytes: CD11b^−^F4/80^−^CD146^−^CD206^−^; liver sinusoidal endothelial cells (LSECs): CD11b^−^F4/80^−^CD146^+^CD206^+^. **c** Cytokine levels in plasma from ePL treated mice (n = 3/group) 4 h after i.v. injection. **d** Scheme of in vivo-in vitro serum testing assay. Cells were supplemented with mouse serum extracted from ePL- or PBS-treated mice. **e** Heatmap obtained from luminescence readout of various transduced cells as indicated. **f** mRNA transcripts expression of immune defense and anti-viral genes in cultured primary macrophages after treatment with 1 mg/mL ePL in vitro. Nanostring nCounter gene expression counts are shown (n = 2/gr). **g** ePL activated STAT1 in macrophages as shown by increased phosphorylation at Tyr701. *N.d*.  not detected

The next step was to uncover the driving forces behind heart targeting and liver detargeting with ePL. Given that more than 90% of the injected dose of ePL accumulates in the liver (Fig. 1), we examined the composition of liver cells targeted by the ePL after a bolus injection in C57BL/6 mice (Fig. 3a). Flow cytometric evaluation of liver cells after the injection of Atto647-labeled ePL indicated that a subpopulation of Kupffer cells (KCs), identified by F4/80^+^CD11b^+^, represented the majority of the ePL uptake in the liver (Fig. 3b, gating strategy in Additional file 1: Fig. S3a). We tested intraperitoneal (i.p.) and intravenous (i.v.) routes of administration because our earlier studies showed that i.p. injections of ePL do not produce heart targeting of nanoparticles and AAVs (data not shown). Other cell types tested (T regulatory cells, F4/80^+^CD68^+^ KCs, hepatocytes, LSECs, and neutrophils, Fig. 3a) [27] were only marginally targeted by ePL. F4/80^+^CD11b^+^ KCs are known to be liver-resident macrophages that secrete various acute response factors including cytokines [28, 29]; therefore, we tested plasma cytokine levels in mice injected with ePL (Fig. 3c). Interestingly, profiling major cytokines using a Luminex 36-cytokine panel showed that ePL did not induce the release of either immunosuppressing or inflammatory cytokines 4 h after i.v. injection (Fig. 3c).

Because F4/80^+^CD11b^+^ KCs are known to secrete factors other than cytokines [30], we hypothesized that the presence of non-cytokine factors in the serum could be attributed to heightened AAV uptake as shown above. To test this hypothesis, we performed in vivo*-*in vitro serum screening assays as depicted in Fig. 3d. Here, C57BL/6 mice were injected with ePL or PBS (normal control) and serum was collected 1 h p.i.. Next, we cultured 5 different cell lines and primary cardiomyocytes obtained from C57BL/6 mice. The cultured cells were treated with collected serum at different doses in the presence of AAV1 carrying a luciferase transgene. Luciferase luminescence was then analyzed 24 h after the treatment and normalized to the total cellular protein. Strikingly, human embryonic kidney (HEK293) and cervical cancer HeLa cell lines (non-muscle, non-cardiac) did not show any significant AAV1 uptake enhancement with ePL serum vs. control normal serum, while cardiomyoblast H9C2 cells and L6 rat skeletal muscle myocytes and myotubes demonstrated robust dose-dependent increases in transduction (Fig. 3e, Additional file 1: Fig. S3b-g). Primary cardiomyocytes showed preferential AAV1 accumulation when treated with the ePL serum, however, only at the lowest dose of the ePL serum. This is likely due to the disruption of the normal cardiomyocyte phenotype by high serum concentrations in culture, as was reported previously. [31, 32]

In order to further elucidate the actions of ePL in macrophages, we incubated ePL with mouse primary bone marrow derived macrophages (BMDMs) in vitro. Fluorescent Atto647-labeled ePL was rapidly engulfed by BMDMs as seen from fluorescence microscopy imaging (Additional file 1: Fig. S3h). In contrast, hepatocyte-like HepG2 cells showed almost no accumulation. Further, ePL drastically increased a number of viral defense genes in cultured BMDMs, including those responsible for viral replication and translational initiation (*Ifit, Stat*), indicating an anti-viral uptake phenotype. This was registered 24 h after ePL incubation in culture (Fig. 3f). Most notably, ePL rapidly increased STAT1 activation manifested in the increase in phosphorylation at Tyrosine 701 [33]. This occurred as fast as 30 min after ePL incubation with BMDMs (Fig. 3g) and persisted for at least 2 h. STAT1 and its activation through phosphorylation are essential for the host immune defense, especially in the context of viral infections [34]. Therefore, it is possible that ePL “primes” the macrophages, KCs, and possibly other cells, to be less permissive to infection by AAV, thus causing the delayed AAV clearance by the immune system.

Collectively, this data suggests that the ePL serum contains yet unidentified factor(s) responsible for paracrine signaling that leads to the drastic increase of AAV and nanoparticle targeting of the heart, and that ePL induces an antiviral-uptake phenotype in macrophages.

### ePL allows for delayed blood clearance of AAV and nanoparticles

The findings shown above only partially explain the enhanced AAV and nanoparticle heart uptake and liver detargeting when injected with ePL. Therefore we examined various factors that may contribute to AAV/nanoparticle targeting to the heart.

**Fig. 4:**
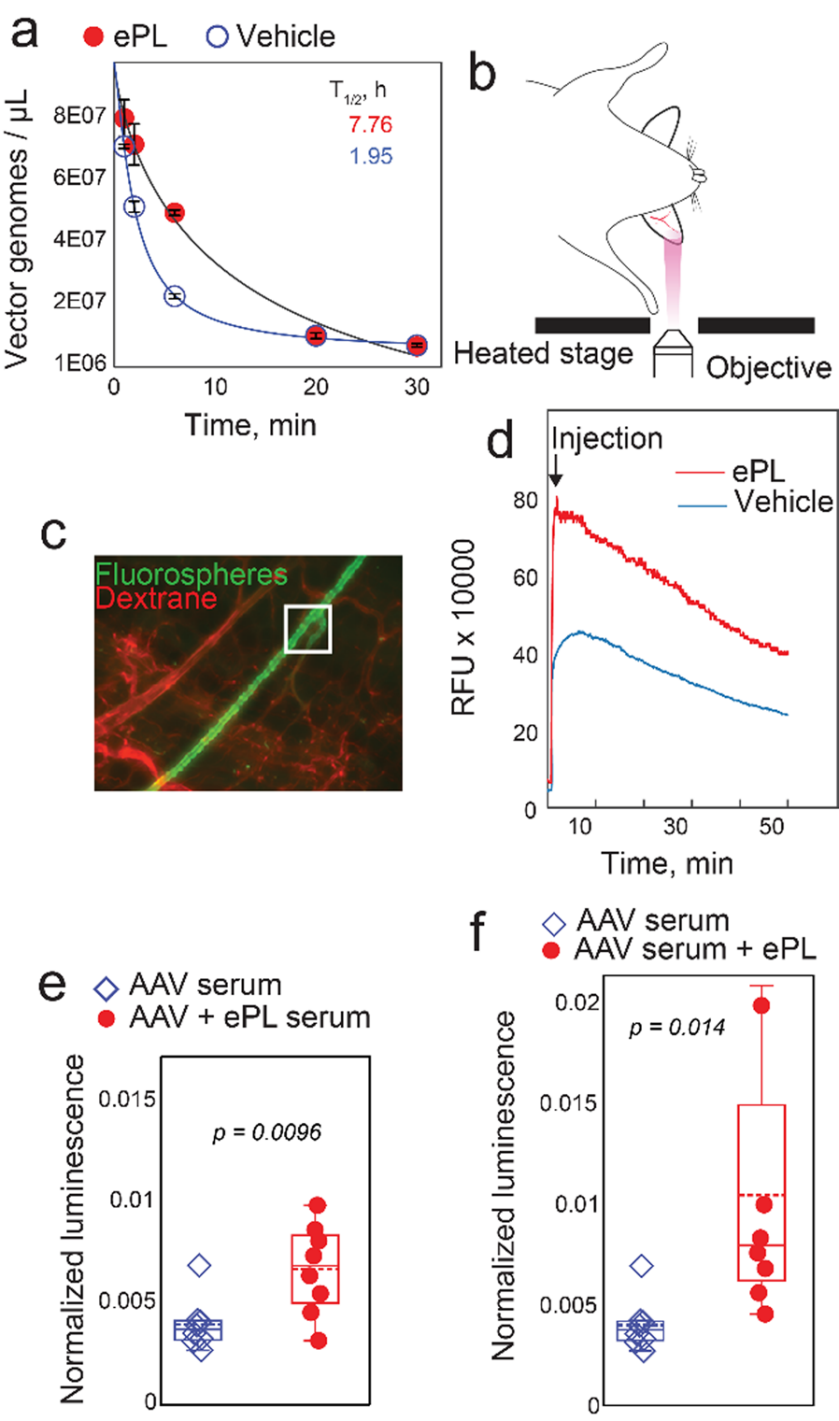
ePL injection changes pharmacokinetics of AAV9 and nanoparticles and acts on neutralizing antibodies. **a** qPCR analysis of blood from AAV9.CMV-GFP injected mouse over 30 h with and without ePL. **b** Ear imaging IVM setup. Intravital microscopy (IVM) enabled blood clearance quantification of model latex nanoparticles with and without ePL pre-treatment. **c** ePL or vehicle PBS was administered i.v. to C57BL/6 mice 15 min prior to injection of latex fluorospheres (green) via catheter. Background staining was accomplished after injection of rhodamine dextran (red). A representative blood vessel imaging is depicted. A white rectangle is shown as a typical region of interest in which signal over time was quantified. **d** Quantification of the fluorescence signal over time from IVM experiments. **e** Lec2 cell transduction assays in the presence of AAV9-injected mouse serum with and without ePL injection. AAV9.CMV.Luc was used to transduce the cells and luminescence values were normalized to total protein content. **f** Lec2 transduction assays as in e, but with ePL added directly into cell culture media

Previous studies on AAV9, a serotype with one of the best-in-class heart transduction efficiencies [19] and the longest systemic circulation [35], have proposed that increased circulation half-life may help overcome slow transvascular AAV transport through the tightly sealed capillary endothelium in the heart, thus efficiently transducing cardiomyocytes by virtue of a substantially longer circulation time [35]. Pharmacokinetic studies using ePL co-administered with 1e10 vg/kg AAV9 demonstrated a 4.0 ± 0.6-fold (p = 0.011) increase in the circulation half-life with ePL as compared to vehicle-injected mice (Fig. 4a).

To determine if the findings on AAV9 pharmacokinetics extend to nanoparticles as well, we performed blood clearance measurements of FluoSpheres latex polystyrene nanoparticles using intravital microscopy (IVM) of C57BL/6 mouse ear microcirculation (Fig. 4b). IVM has been previously used to measure clearance of various nanoparticles in several mouse models with success [36]. This method is non-invasive and allows for blood clearance recording in real time. FluoSpheres (1 mg/kg) were injected just after the bolus ePL at 30 mg/kg i.v. and the fluorescence was measured over 1 h. The decay of fluorescence intensity in the mouse ear vein was recorded and plotted against time (Fig. 4c, d). The data indicate that ePL injection significantly delayed the blood clearance of FluoSpheres, especially very early (< 1 min) after the injection.

To explore the possible mechanism behind such delayed blood clearance of AAV9 and FluoSphere nanoparticles, we looked into potential involvement of neutralizing antibodies (nAbs). Pre-existing nAbs impair AAV persistence in circulation by opsonization [37, 38]. To investigate whether ePL has the ability to reduce or prevent nAbs production, we designed an in vivo-in vitro assay that leveraged inherent immunogenicity of AAV9 to produce high levels of nAbs after a single injection [39, 40]. Two separate experiments were conducted. First, a bolus i.v. injection of AAV9 at 1e10 vg/kg in C57BL/6 mice with or without 30 mg/kg of ePL, followed by serum collection 30 days later produced AAV9-specific nAbs-enriched serums. These serums were mixed with AAV9 at MOI 50,000 in culture medium followed by the incubation with Lec2 cells, a cell line that expresses sialylated glycans on the surface, which are known targets for the AAV9 intracellular entry. The results show that co-injection of AAV9 with ePL produced significantly less nAbs as evidenced by the increased AAV9 infectivity of Lec2 cells when the corresponding serum was added (6.7E-3 ± 8E-4 for AAV9 + ePL serum vs. 4.0E-3 ± 5E-4 for AAV9 serum, p = 0.0096, Fig. 4e). Second, using the serum from AAV9-only injected mice, we incubated the Lec2 cells in the presence of ePL, spiked directly in the culture medium. Strikingly, this similarly increased AAV9 infectivity (1.0E-2 ± 2E-3 for ePL-spiked cells vs 4.0E-3 ± 5E-4 for AAV9 serum only, p = 0.014, Fig. 4f), suggesting that ePL may have the ability to directly prevent AAV9 neutralization by nAbs.

Collectively, these data suggest that ePL improves the blood residence time of AAV and nanoparticles by acting on nAbs as at least one of the modes of its action.

### ePL paracrine signaling in cardiomyocytes enables heart targeting

**Fig. 5:**
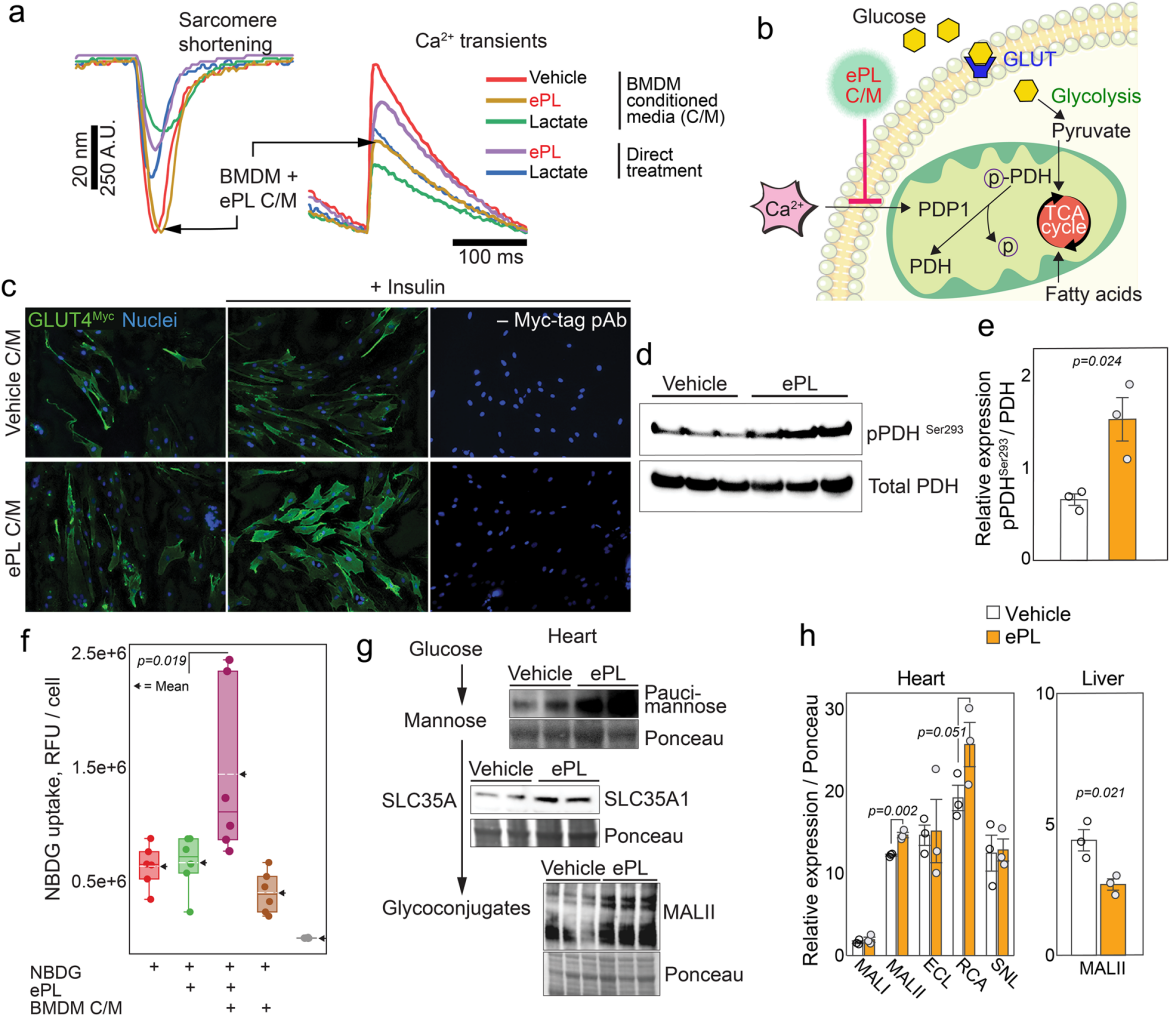
ePL induces paracrine signaling in the heart. **a** Contractility and intracellular Ca^2+^ in primary mouse cardiomyocytes in response to ePL or conditioned media (C/M) from bone marrow-derived macrophages (BMDM) pre-incubated with ePL. **b** Schematic demonstrating ePL C/M effect on calcium uptake and the subsequent signaling cascades resulting in increased glucose uptake. **c** GLUT4 translocation assays in rat L6 myotubes expressing Myc-tagged GLUT4. **d** Western blot of heart lysates from PBS- or ePL-treated mice probing for expression of pPDH Ser293. **e** Quantification of immunoblot in d. **f** NBDG (fluorescently-labeled glucose) uptake in primary cardiomyocytes using ePL alone or conditioned media (C/M) from ePL-treated BMDMs. **g** Western blot analysis of heart lysates from ePL-treated mice, showing higher expression of paucimannose, SLC35A1, and glycoconjugates detected by lectin MALII. **h** Quantification of immunoblots in g, with additional data on liver expression (see Additional file 1: figures). n = 3 animals per group for in vivo experiments and n = 6 for in vitro experiments in isolated cardiomyocytes

Because ePL is sequestered rapidly by macrophages in vitro and KCs in vivo and the serum from mice injected with ePL aids in the AAV uptake in muscle/cardiac cells, we hypothesized that one of the ways ePL exerts its actions in the heart could be through communication between macrophage secretome and heart cardiomyocytes. To test this, we performed a series of experiments to probe paracrine signaling in the heart induced by ePL (Fig. 5). We isolated primary mouse cardiomyocytes from C57BL/6 mice and conditioned medium (C/M) from BMDMs cultured in serum-free medium and treated with ePL, PBS vehicle control, and lactate, which is a product of ePL biodegradation. Next, using cardiomyocytes pre-loaded with fluorescence indicators, we conducted a series of treatments using various C/Ms while simultaneously recording calcium levels (Ca^2+^) and the cell length of single cardiomyocytes through quantitative video microscopy. Notably, ePL C/M significantly decreased the amplitude of cytosolic Ca^2+^ transients without significant changes in cardiomyocyte contraction (Fig. 5a, Additional file 1: Fig. S4a, b). Direct treatment of cardiomyocytes with ePL or lactate did not have the same effect, or its magnitude was substantially lower relative to untreated BMDM C/M (Additional file 1: Fig. S4a, b).

It has been previously proposed that the decrease in Ca^2+^ entry in cardiomyocytes may be a driver of increased glycolysis and glucose uptake in the heart (Fig. 5b) [41]. Indeed, cardiomyocyte in vitro glucose uptake was increased with ePL C/M treatment (Fig. 5f). Glucose uptake is dependent on the expression of GLUT transporters in all cells. We tested whether muscle-specific GLUT4 transporter expression is altered after ePL C/M treatment. To accomplish this task, we chose a well validated, genetically engineered L6 cell line constitutively expressing myc-tagged GLUT4. Immunostaining with anti-myc antibodies in these cells allows for sensitive tracking of the GLUT4 expression. As expected, GLUT4 was highly upregulated in these cultured muscle L6 myotubes upon ePL C/M treatment (Fig. 5c). The expression of GLUT4 was time-, ePL C/M dose- and insulin-dependent (Additional file 1: Fig. S4c, d).

To further investigate the effects of reduced Ca^2+^ uptake by ePL, we looked into metabolic enzymes that regulate glucose metabolism and are Ca^2+^-dependent. Pyruvate dehydrogenase (PDH) is the enzyme that catalyzes the transformation of pyruvate to acetyl-CoA, however, phosphorylated PDH (pPDH), including at serine 293, inhibits this catalytic action [42]. Notably, pPDH dephosphorylation is catalyzed by a Ca^2+^-sensitive phosphatase PDP1 (Fig. 5b). It has been previously shown that Ca^2+^-mediated dephosphorylation of pPDH in cardiomyocytes decreases glucose oxidation and promotes glycolysis, without changes in cardiomyocyte contraction [41]. Given these facts and the results above, we tested whether pPDH levels change in the heart after injection of ePL in C57BL/6 mice. Indeed, the treatment with ePL resulted in significantly higher levels of pPDH as compared to vehicle PBS-injected control (1.54 ± 0.23 for ePL vs. 0.68 ± 0.6 for PBS, p = 0.02, Fig. 5d, e). This data is suggestive of cardiomyocyte “reprogramming” in response to ePL C/M treatment, causing cardiomyocytes to ramp up their glucose consumption, likely due to dampened dephosphorylation by PDP1.

Enhanced glucose uptake in cardiomyocytes could be partially responsible for glycosylated VLNPs uptake in the heart in vivo. However, even though many AAVs are highly surface-glycosylated [43, 44], AAV tissue uptake through GLUT glucose transporters is largely unknown. Rather, tissue protein glycosylation seems to have a significant role in predicting AAV organ tropism, which is governed by the expression of various N- and O-glycoprotein glycans on the cell surface, serving as receptors for the majority of the AAV serotypes [45–47]. Protein glycosylation is partially driven by glucose-derived mannose that is directly used for glycoconjugate synthesis. To test whether ePL plays a role in tissue glycoconjugate expression, we injected a bolus ePL at 30 mg/kg or PBS vehicle control in C57BL/6 mice and extracted whole hearts 1 h later. The heart-derived proteins were analyzed by Western blotting immunoprobing for key molecules involved in glycoconjugate synthesis (Fig. 5g). First, pauci-mannosylation was assessed using antibodies against paucimannose, which detect the posttranslational modification of proteins by simple mannose units. ePL injection significantly increased the levels of paucimannose in the heart, suggestive of glucose-to-mannose conversion in the first step of glycoconjugate synthesis [48]. Next, in the same heart lysates, we probed for an CMP-sialic acid transporter, also known as CMP-Neu5Ac, which is a key transporter protein in cellular sialylation (integral component of glycoproteins) [49]. Notably, SLC35A1 was also highly upregulated in the hearts of ePL-treated mice (Fig. 5g). Finally, we screened for diversity of glycoconjugates in the same heart lysates by probing with various lectins that detect specific oligomannose-rich glycoproteins (Fig. 5h, Additional file 1: Fig. S4e). The cardiac proteins from ePL-injected animals demonstrated statistically significant increase in binding to MALII lectin (*Maackia Amurensis*) that binds to sialic acid in an (α-2,3) linkage (14.69 ± 0.33 for ePL vs. 12.24 ± 0.11 for vehicle, p = 0.002) [50]. Similarly, RCA lectin (*Ricinus Communis Agglutinin)*, which binds to galactose or N-acetylgalactosamine also demonstrated enhanced affinity to proteins from ePL-injected heart extracts (Fig. 5h, Additional file 1: Fig. S4e). Conversely, liver tissue lysates showed a significant reduction in binding to MALII (2.7 ± 0.2 for ePL vs. 4.4 ± 0.4 for vehicle, p = 0.2, Fig. 5h, Additional file 1: Fig. S4f), suggesting that such differential glycoprotein expression in the heart vs. the liver could be a consequence of heart targeting and liver detargeting by AAV. According to literature, the tissue expression of N-linked α-2,3 sialic acid predicts high tropism for AAV1, whereas galactose expression predicts tropism for AAV9. [51]

Collectively, these data suggest that paracrine signaling in the heart via serum-derived factors in ePL-injected mice reprogram heart metabolism towards enhanced glucose consumption, which explains the heightened uptake of the glycosylated VLNPs. At the same time, ePL-induced glucose uptake in the heart facilitates cardiac protein glycosylation, pointing to the possible mechanism by which ePL modulates the affinity of AAV to the heart.

### ePL increases glucose uptake in the heart in vivo

**Fig. 6:**
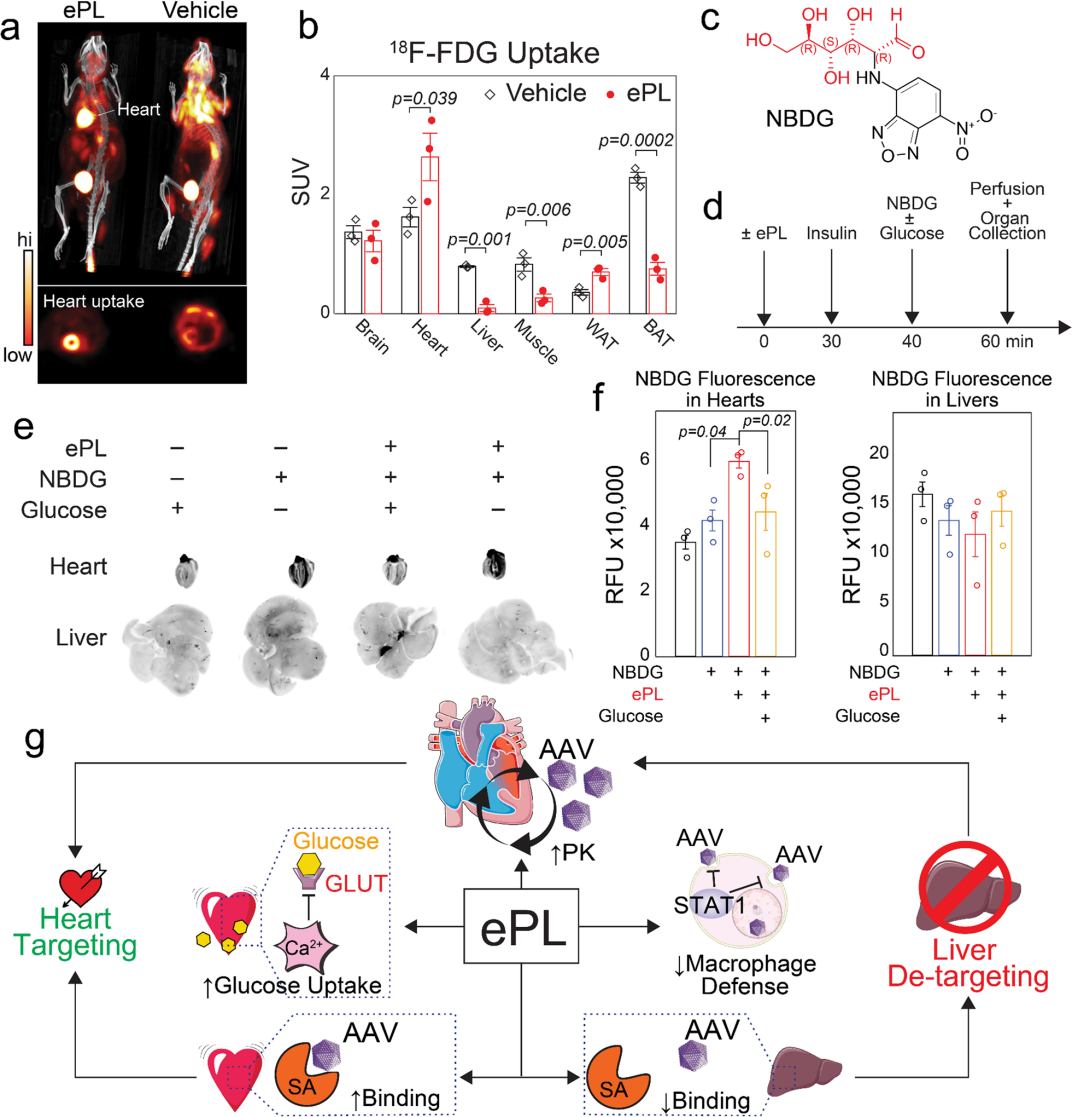
ePL increases heart glucose uptake in vivo. **a** Representative PET/CT images from experiments on ^18^F-FDG tracer uptake in mice pre-injected with ePL or PBS vehicle control. **b** Quantification of ^18^F-FDG tracer uptake in different organs, using the standardized uptake values (SUV). **c** Molecular structure of fluorescent glucose analog, 2-(7-Nitro-2,1,3-benzoxadiazol-4-yl)-D-glucosamine (NBDG). **d** Injection schedule of NBDG and ePL in experiments with in vivo glucose uptake and competition with unlabeled glucose. **e** Whole organ fluorescence imaging after ePL-NBDG experiments as depicted in d. **f** The fluorescence in the hearts of animals from e was quantified after background subtraction and normalized to the organ weight (n = 4 mice per group). **g** Proposed mechanisms of action of ePL. ePL increases circulation half-life of AAV, glucose heart uptake and reduces liver off-target effects

The previous experiments demonstrate increased glucose uptake by isolated cardiomyocytes in response to ePL C/M. To test whether this occurs in vivo, the whole body glucose uptake was assessed using fluorodeoxyglucose-^18^F (FDG)-positron emission tomography (PET). A single dose of ePL significantly increased ^18^F-FDG tracer uptake in the heart just 1 h after ePL administration, as compared to vehicle-injected animals (1.6 ± 0.2-fold change, p = 0.039, Fig. 6a, Additional file 1: Fig. S5a, quantified in Fig. 6b). This occurred concomitantly with no changes in the FDG uptake in the brain; however, the uptake in the liver—another major glucose-consuming organ—was significantly reduced (7.0 ± 0.1-fold change, p = 0.001, Fig. 6b).

In light of the data above, the increased ^18^F-FDG tracer uptake in the heart could be attributed to its delayed blood clearance in response to ePL (Fig. 4d). In that case, the signal seen in the heart could be explained by the ^8^F-FDG distribution in the blood pool, without necessarily heart-specific uptake. However, the blood clearance of nanoparticles and viruses is governed by drastically different forces than that of the small molecules such as FDG or glucose. Nevertheless, to rule out that possibility, the in vivo heart uptake of the fluorescent analogue of glucose, NBDG (Fig. 6c), was studied with and without ePL. In addition, the injections with ePL and NBDG were carried out in the presence of the large excess of unlabeled glucose, as a competitor for heart uptake (Fig. 6d). The data showed that even after extensive organ perfusion, NBDG fluorescence was highly localized in the heart of animals pre-injected with ePL (4.16E4 ± 0.32E4 for vehicle vs. 5.98E4 ± 0.20E4 for ePL, p = 0.04, Fig. 6e, quantified in Fig. 6f). However, it was diminished in the competition experiments with unlabeled glucose (5.98E4 ± 0.20E4 for ePL vs. 4.43E4 ± 0.57E4 for ePL + glucose, p = 0.02). Liver (Fig. 6f) and other organs such as lung (Additional file 1: Fig. S5b, c) were somewhat detargeted by NBDG when ePL was injected.

The data above suggest an opposing action of ePL on glucose uptake in the heart and liver. These data are supportive of the preferential heart delivery of vehicles/entities that are glycosylated and the mechanisms related to tissue glycosylation described above (Fig. 5g, h). Moreover, the enhanced uptake of NBDG with ePL is yet another example of an entity that can be delivered with high selectivity to the heart, i.e. a small molecule. A fluorophore in this case, but potentially, any pharmacologic agent if glucose-conjugated without the loss of therapeutic activity, is likely to improve heart targeting with ePL.

## Discussion

We discovered a novel enhancer, ePL, that enables liver de-targeting and heart targeting. These properties make it a suitable enhancer for chemical or biological modalities that target cardiac pathologies.

ePL has several advantages that can be leveraged for improving upon existing or discovery of novel efficacious pharmacological treatments for cardiac pathologies. One advantage of ePL is that it is an enhancer that doesn’t require co-formulation with the chemical or biological modalities it is enhancing. Another advantage is its potential to be compatible with any AAV vector: any new AAV vector with improved cardiac tropism would still be further enhanced by pre-treatment with ePL. Additionally, ePL is safe even at high doses. Finally, the efficacious doses required for cardiac-targeting pharmacological agents are likely to be lower when used with ePL vs. alone.

The application of ePL has two requirements. First, ePL must be administered i.v. before administering a therapeutic modality. Second, a modality that is enhanced by ePL must have proclivity to interact with the glucose-GLUT axis; for example, a nanoparticle decorated with glucose (VLNP), an AAV vector that recognizes glycosylated surfaces of cells, or a small molecule conjugated to glucose.

We tested two modalities, AAVs and glucose-decorated nanoparticles (VLNP) with ePL and discovered the overarching mechanism of action of ePL that includes the RES blockade (KCs in the liver) and the glucose uptake in the heart. Additionally, ePL increases circulation half-life of each modality tested. In the case of VLNPs, the mechanism relies primarily on the interaction of surface glucose molecules with the GLUT4 transporter on the surface of cardiac cells to stimulate the uptake of the nanoparticles by the heart. In case of AAVs, ePL exerts additional actions. It promotes cardiac cell sialylation recognized by AAVs and suppresses anti-AAV neutralizing antibodies.

The ePL-driven enhancement of AAV uptake in the heart is an especially intriguing discovery in our study as it opens an opportunity to drastically reduce an effective dose of an AAV vector to treat genetic cardiomyopathies. Two ePL-AAV-specific mechanistic findings are worth highlighting (Fig. 6g).

First, the glucose uptake upregulation in the heart in response to an ePL injection increases the production of glycoconjugates. It has been speculated that AAV capsids have an affinity to highly sialylated cardiac tissue proteins which drives the uptake of AAV in the heart [19]. Moreover, high glucose uptake in the heart correlates with increased insulin receptor (IR) expression [52]. Even though we didn’t specifically investigate the expression of IR in the heart, it has been theorized that IR may serve as a co-receptor for AAVs [53] by virtue of its similarity to other known AAV co-receptors, including the epidermal growth factor receptor and the platelet-derived growth factor receptor [19], both of which are responsible for recognition of some AAV serotypes and for their uptake inside the cell.

The second notable mechanistic finding is that ePL increases the half-life of a circulating AAV. We suggest two reasons for this increase. The first reason is the “blocking” of the liver where ePL causes Kupffer cells to activate their viral defense mechanisms. Since the liver is a major sink of AAV when administered without ePL, this blocking allows AAV to remain in circulation longer. The second reason is the ePL effect on anti-AAV neutralizing antibodies (nAbs). We have shown that ePL blocks pre-existing nAbs, and, strikingly, decreases the production of nAbs. Pre-existing nAbs typically impact AAV circulation time by forming an immune complex with AAVs that is readily taken up by the liver. Since ePL decreases production of nAbs, this technology could potentially enable multiple doses of AAV to be administered to a patient. Multiple AAV doses are currently not possible because the second dose would result in a severe immune response rendering the second AAV dose ineffective. Although our work did not aim to enable repeat doses of AAV, this is a fortuitous finding.

To the best of our knowledge, there is no precedent of heart targeting with an enhancer molecule like ePL. Therefore, the ePL approach represents a novel platform for delivering therapeutic agents to the heart. Specifically, it opens up an opportunity to more effectively deliver genetic therapies to the heart to treat previously intractable genetic cardiomyopathies. Future directions for the ePL approach include translation of these findings to non-human primates and testing a selected therapeutic agent in a mouse model of a genetic cardiomyopathy.

## Study limitations

This work is not devoid of important limitations. Even though we speculated that targeting modalities other than AAVrh.74, AAV1, VLNPs and model latex nanoparticles are likely to be enhanced by ePL to improve their affinity to the heart, we did not specifically investigate this. An enormous number of AAV serotypes, nanoparticles of various kinds, dendrimers, biologics and many more, have been developed to date, which opens an exciting opportunity for further testing, but beyond the scope of this study. The mechanisms of action of ePL are multi-prong and are not limited to one defined action. We do not exclude the possibility that other mechanisms may be discovered in the future. While we have yet to understand the nature of serum-derived factors responsible for ePL-mediated heart targeting, knowing their identity is unlikely to result in a standalone biologic agent that would be as effective as ePL. Rather, the fine-tuning physicochemical characteristics of the existing ePL formulation is essential to optimize its efficacy and improved performance for human translation.

## Methods

### General

All chemicals were of the highest grade of purity (analytical grade) and purchased from Millipore Sigma or Fisher Scientific, unless specified otherwise. Cell culture supplies including culture media, fetal bovine serum, antibiotics and other related supplies were purchased from Thermo Fisher Scientific unless specified otherwise. Adeno-associated viral vectors (serotypes 1 and 9) encoding eGFP or firefly luciferase under the control of human cytomegalovirus (CMV) promoter were purchased from Signagen (SL100803, SL101493, SL100840, SL101494). AAVrh.74-CMV-eGFP vectors were obtained from Nationwide Children's Hospital Vector Core (Lot# TT667-1P). All viral vectors were obtained at ≥ 1e13 vg/mL determined by the vendor using qPCR or droplet-digital PCR. Lec2 cells were obtained from American Type Culture Collection (ATCC, CRL-1736) and cultured according to the vendor’s instructions. L6-GLUT4myc rat myoblast cell line was obtained from Kerafast (ESK202-FP), cultured and differentiated into myotubes as suggested by the distributor. HepG2 cells were obtained from ATCC (HB-8065). Primary bone marrow-derived macrophages were obtained, cultured and differentiated as previously described by us [55, 56]. Primary cardiomyocytes were isolated from 6 to 8 weeks old C57BL/6 mice and cultured as previously described by us [57, 58]. Cytokine analysis was performed by Eve Technologies (Calgary, AB Canada) using mouse discovery multiplex Luminex-based assays.

### Animals

Male, C57BL/6 mice (8–10 weeks of age) were purchased from The Jackson Laboratory (stock 000664) and kept in AAALAC-accredited facilities at Case Western Reserve University. All experiments were conducted according to IACUC-approved protocol (protocol# 2016-0273) and IBC protocol (IBC-2021-406) for work with AAV in animals. Mice were housed five per cage and allowed to acclimate in the vivarium for 1–2 weeks before the start of the experiments. Throughout the experiments, the animals were kept on a 12:12 h light–dark cycle at 22 ℃, and both diet and water were provided ad libitum. The animals were used at 4–10 per group for all delivery experiments and at 3 per group for positron emission tomography imaging. The number of animals per treatment condition is indicated in the manuscript’s legends.

### ePL Synthesis and characterization

The ePL was synthesized from PLGA (Millipore-Sigma, 900,316) through nanoprecipitation. Briefly, the PLGA was dissolved in acetonitrile (organic phase) at 10 mg/mL which was then added dropwise into 0.1% PVA aqueous solution (aqueous phase), while continuously stirring at 1000 rpm. The organic phase was partially evaporated in the chemical fume hood while stirring in the open container. Residual acetonitrile, PVA, and excess of water were removed through tangential flow depth filtration using counter flow columns (Repligen C02-E750-10-S) driven by peristaltic pumps. After concentrating to 10–13 mg/mL, sterile filtration through 0.45 µm filter, and buffer exchange to 10 mM PBS, the size of ePL was measured with Malvern Zetasizer Nano series ZS. Fluorescent ePL was synthesized by the addition of ATTO647N-conjugated PLGA polymers, which were obtained as follows. Amine end-capped PLGA (PolySciTech, AI062) and ATTO647N-NHS fluorophore (Millipore-Sigma, 07376) were mixed in dry dichloromethane at 1:1 molar ratio and reacted at room temperature for 3 h with vigorous shaking. The resulting ATTO647N-conjugated PLGA were then washed twice with cold methanol and precipitated by centrifugation at 1,000 g for 10 min. The residue lyophilized overnight and reconstituted in acetonitrile. During regular ePL synthesis described above, the fluorescent polymer was added at 5% (w/w) to non-fluorescent PLGA as an organic phase.

### Biodistribution of ePL

ePL was labeled with europium (Eu) cryptate to allow for time-resolved fluorescence imaging (TRFI) as previously described by us [59]. TRFI is a technique that allows for higher signal-to-noise ratios when imaging whole organs as it circumvents the detection of endogenous fluorescence (autofluorescence) ubiquitous in any organ but especially pronounced in liver and digestive tract. C57BL/6 male mice of 8 weeks of age were switched on Alfalfa free diet (Envigo, 2916, now Inotiv) for 2 weeks prior to the imaging. The C57BL/6 mice were fasted overnight (12 h) and injected with 30 mg/kg of Eu-labeled ePL. The injection was a bolus i.v. through a retro-orbital plexus. One hour after the injection, the animals were euthanized and perfused in a reduced light environment through the left ventricle with cold PBS-citrate buffer (10 mM PBS containing 11 mM trisodium citrate, pH 7.2) for at least 5 min. The major organs were then excised and immediately imaged using TRFI on a Molecular Devices i3 reader equipped with TRF module. In some experiments, the imaging was performed 6 h after the injection to detect ePL excretion in major gastrointestinal tract organs.

### General methodology for ePL testing

Sterile-filtered formulation of ePL at 10–13 mg/mL was injected into the right eye via retro orbital injection at a 30 mg/kg dose, immediately followed by the injection of VLNPs, AAVs or NBDG into the left eye. In some experiments, and as indicated in the manuscript, a delay of 15–30 min between the injections was implemented. VLNPs were administered at 10 mg/kg (based on FluoSpheres weight), AAVs were injected at ~ 2.5e10 vg per mouse or 1.0e + 09 vg per kg, and NBDG (Cayman Chemical, 11046) was injected at 50 mg/kg (solution in PBS). In short term experiments, the mice were euthanized 2 h post injection (p.i.) using isoflurane or pentobarbital overdose followed by the extensive perfusion with PBS-citrate buffer (10 mM PBS containing 11 mM trisodium citrate, pH 7.2) through a left ventricle using gravity perfusion apparatus at a rate of 2 mL/min for 5 min. For long term AAV protein expression experiments, the mice were euthanized 30 days p.i. and the perfusion was omitted. Heart, liver and other organs (as indicated in the manuscript) were then excised and either subjected to the fluorescence imaging (for VLNPs and NBDG) or flash-frozen in liquid nitrogen (for AAVs). In some experiments, the organs were immersed into 4% paraformaldehyde overnight at room temperature followed by embedding into paraffin blocks using standard dehydration-embedding technique. The expression of VLNP or NBDG fluorescence in whole organs was detected using imaging with Azure C400 imager. VLNP fluorescence and eGFP expression in heart and liver sections was detected using immunofluorescence microscopy as described below. Frozen heart, liver and other organs were pulverized in polycarbonate vials (OPS Diagnostics, PCRV 04-240-10) at a liquid nitrogen temperature using SPEX GenoGrinder 2010. The 50–100 mg of the pulverized tissue was then extracted with a lysis buffer and subjected to immunoblotting as described in Supplementary Information. The 20–50 mg of the pulverized tissue was extracted using the extraction buffer from Macherey–Nagel ​​NucleoSpin Tissue kit (Takara Bio, 740,952.250) followed by the DNA isolation according to the manufacturer’s instructions and the analysis by qPCR.

### Statistics

For all experiments, data was first tested for normality using the Shapiro–Wilk test and for equality of variances using Bartlett's test. If it was determined that the normality and equality of variances are satisfied (p ≤ 0.05), the group means were compared using Student’s t-test (for 2 groups) or ANOVA with Tukey’s *post-hoc* test (> 2 groups). In some experiments with > 2 groups, pairwise t-test was applied with Holm *post-hoc* test. The particular statistical analysis employed is indicated in the figure legends of the main manuscript. For non-normally distributed data or data with unequal variances, nonparametric Mann–Whitney U-test (2 groups) was used. The statistical analysis was performed using R version 4.3.0 (“Already Tomorrow”) or later. Graphs and plots were created using Plotly Chart Studio or with R packages tidyverse/ggplot2. The results are presented as mean with standard error of mean (SEM). The SEM values are displayed as error bars in the figures or values following plus-minus sign in the main text.

### Supplementary Information

**Additional file 1. ** Supplementary Figures and Methods.

## Data Availability

Not applicable.
