# Supplementary material for: Engineered nanoparticles promote cardiac tropism of AAV vectors
Source: J Nanobiotechnology. 2024 May 3;22:223. doi: 10.1186/s12951-024-02485-6 (PMC11067271; doi:10.1186/s12951-024-02485-6)
Supplement: Supplementary file 1 — Additional file 1. Supplementary Figures and Methods. [file 12951_2024_2485_MOESM1_ESM.docx]

Supplementary Information for

**Engineered nanoparticles promote cardiac tropism of AAV vectors**

Lauren Switala ^1,2^, Lin Di ^1,2^, Huiyun Gao ^1^, Courteney Asase^1^, Matthew Klos^3^, Palanivel Rengasamy^1^, Daria Fedyukina^4^, Andrei Maiseyeu^1,2^†

1 Department of Medicine, School of Medicine, Case Western Reserve University, Cardiovascular Research Institute, 2 Department of Biomedical Engineering, Case Western Reserve University, 3 Department of Pediatrics, Case Western Reserve University, 4 Bioheights LLC (Current affiliation: Advanced Research Projects Agency for Health, ARPA-H).

†Correspondence should be addressed to Andrei Maiseyeu; E-mail: axm1079@case.edu

**Content:**

Supplementary Figure 1-5

Supplementary Methods

Uncropped Western Blotting Images

Citations

# Supplementary Figures


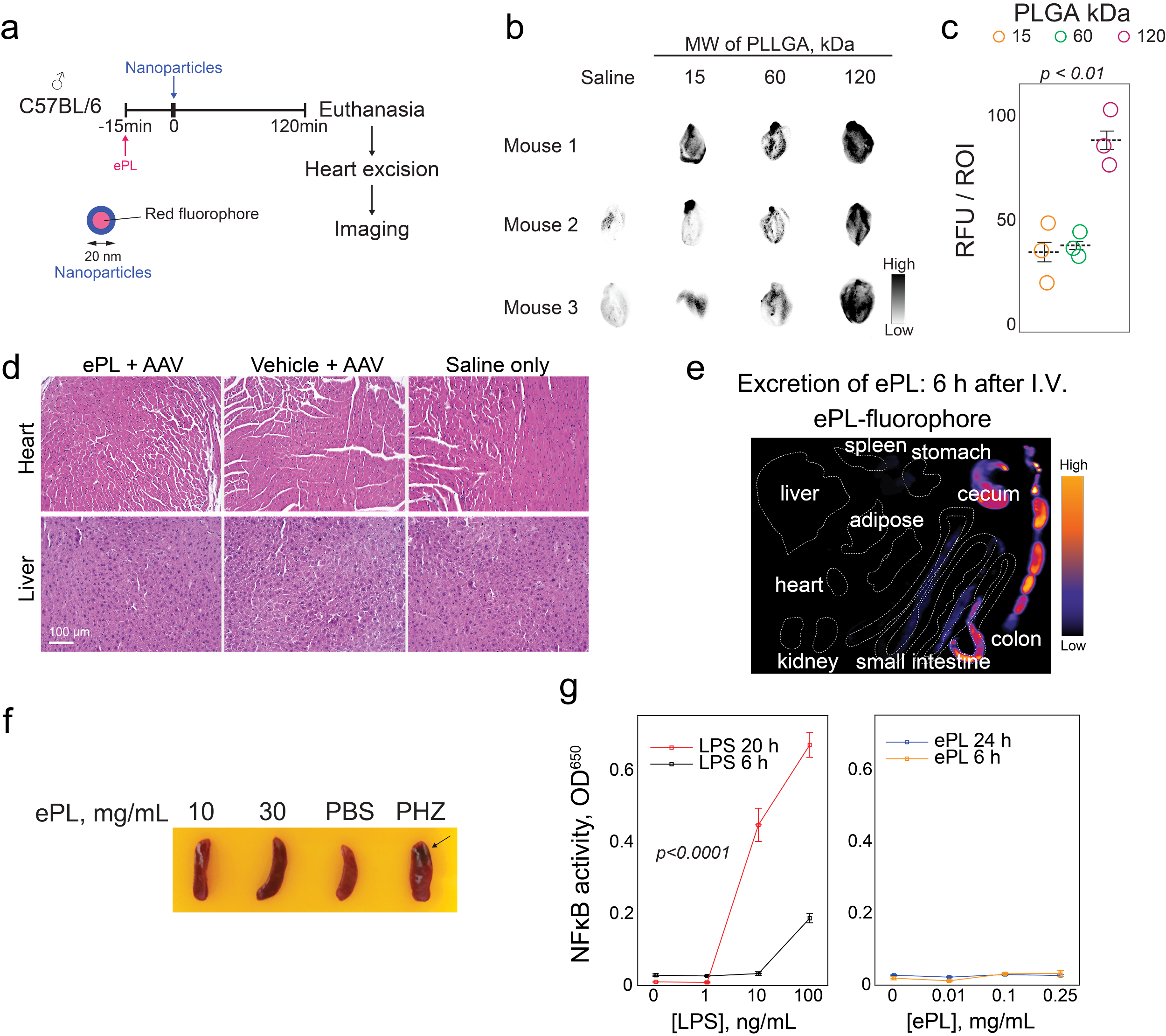


**Supplementary Fig. 1.** a) Experimental schematic for testing nanoparticle formulations injected after various PLGA nanoparticles. b) Screening of three different PLGA formulations on their ability to enhance nanoparticle uptake in the heart. c) Quantification of images in b. d) Histology of heart and liver after co-injection of ePL with AAV1. e) Biodistribution of ePL 6 h p.i, showing excretion in gastrointestinal organs, likely through hepatobiliary excretion mechanism. f) Spleen gross pathology was performed 24 h after ePL injection. Hematotoxin phenylhydrazine (PHZ)[^1,2^](https://paperpile.com/c/11UBca/a8dJo+kAYx8) served as a positive control in this experiment that showed spleen enlargement with signs of splenic infarct (arrow). g) ePL is an endotoxin-free formulation. ePL was tested in cell-based colorimetric assay for the detection of biologically active endotoxin. HEK-blue cells engineered to become extremely sensitive to LPS leading to the activation of NF-kB, were incubated with ePL at different concentrations and two time points (left). LPS was used as a positive control (right).


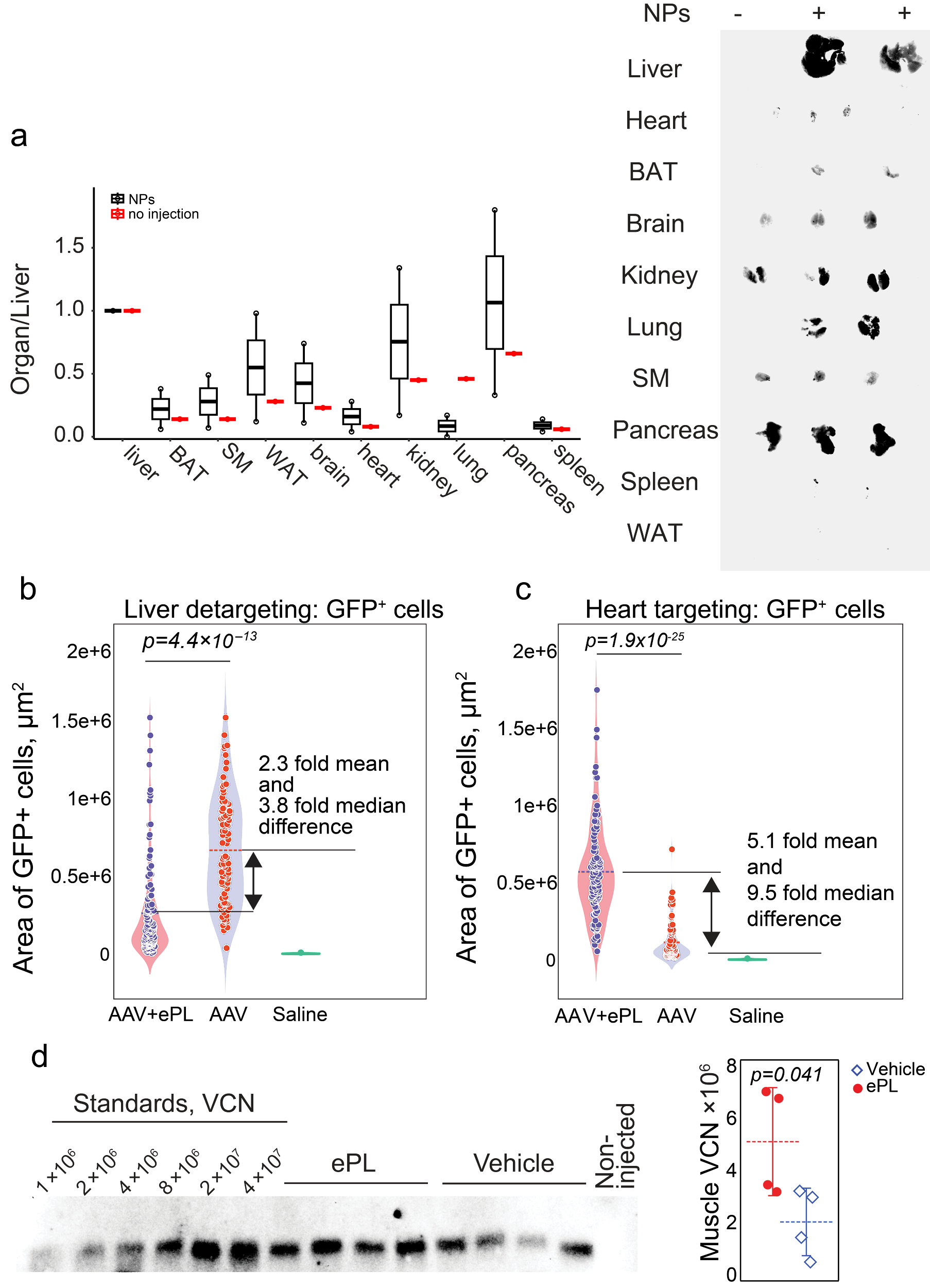


**Supplementary Fig. 2.** a) Biodistribution imaging in various organs of fluorescently-labeled NP 1 h after i.v. administration (no ePL administered), and non-injected control organs (left). Fluorescent intensity is quantified and normalized to organ fluorescence from non-injected animals. b) Quantification of immunofluorescence (IF) in the liver of mice treated with AAVrh74.CMV.eGFP. c) Quantification of IF in the heart of the same mice as in b. d) Southern blot of muscle from mice injected with AAVrh74.CMV.eGFP with and without ePL. Quantification is presented on the right side of the blot. VCN = vector copy number.


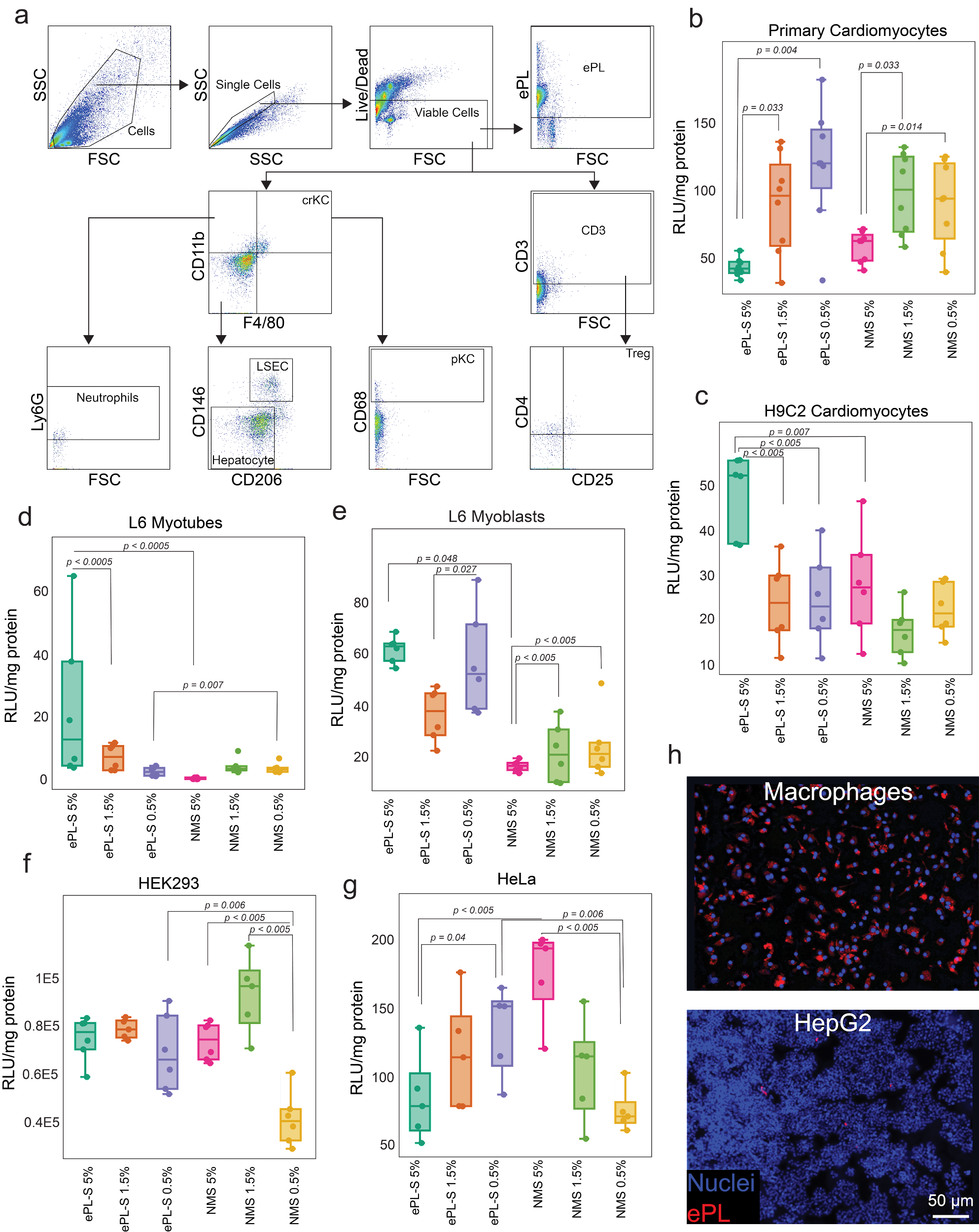


**Supplementary Fig. 3.** a) Representative flow cytometry dot plots showing liver cell gating strategy. (b-g) Transduction of AAV in different cell types cultured with serum extracted from ePL-injected mice (n = 4). Individual cell types summarized in heat map in Fig. 3c of the main manuscript. Note that n value changes between cell types; primary cardiomyocytes n=8, H9C2 n= 6, L6 myotubes n= 6, L6 myoblasts n=6, HEK293 n=6 (ePL-s 1.5% n=5, NMS n=5), HeLa n=5. h) Representative fluorescent microscopy image of *in vitro* ePL uptake in macrophages and hepatocyte-like HepG2 cells.


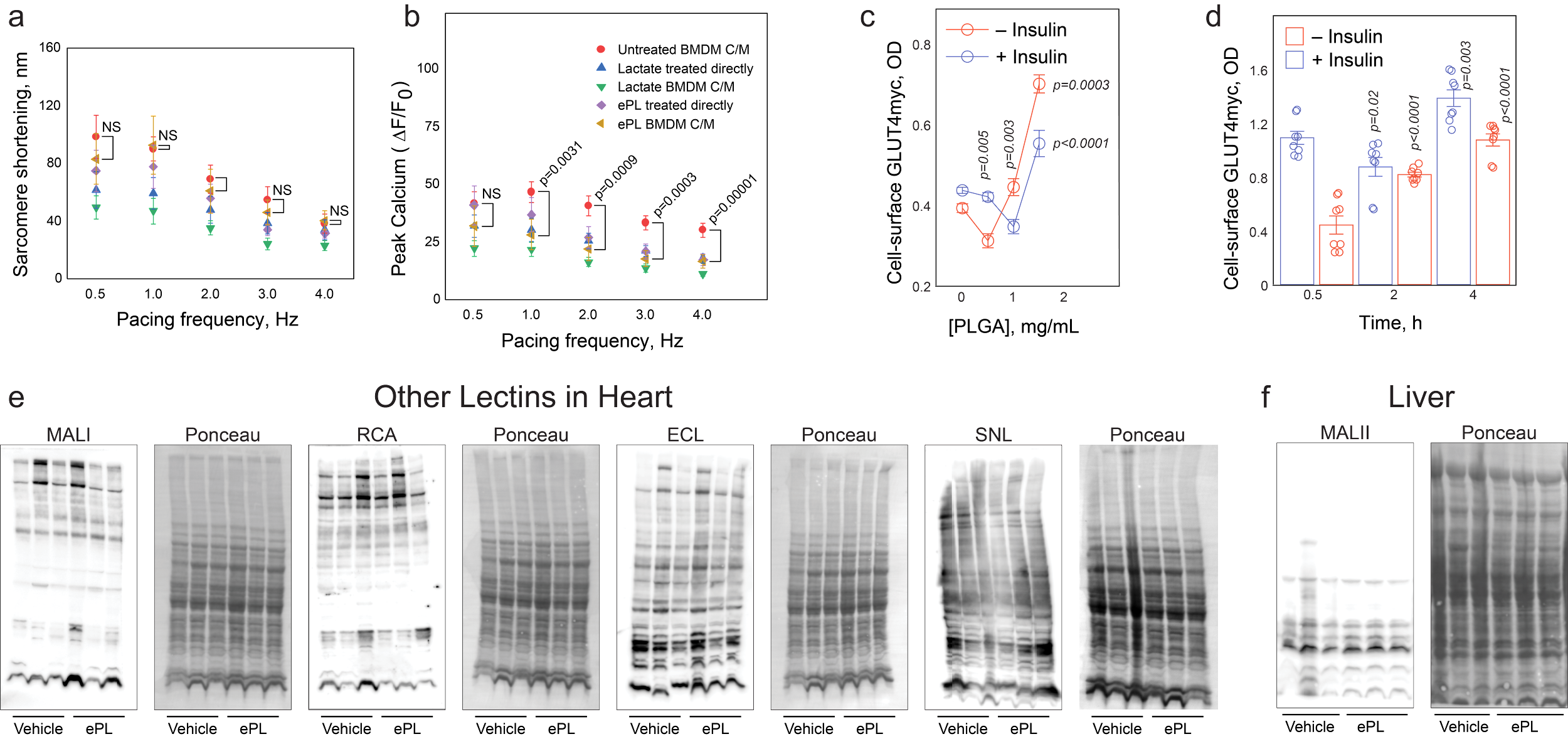


**Supplementary Fig. 4.** a,b) Quantification of sarcomere shortening and calcium influx in primary cardiomyocytes in response to different cell conditioned mediums (C/M) as indicated. c) L6 myotubes expressing myc-tagged GLUT4 were incubated with C/M obtained from BMDMs treated for 4 h with different concentrations of ePL. GLUT4 expression in these cells pulsed with and without insulin was then tested using a myc-tag colorimetric detection. P values are from pairwise t-test with Holm correction and vs. C/M from untreated BMDMs (0 mg/mL). d) GLUT4 expression in similar experiment as in c, however with C/M obtained from BMDM’s incubated with 1 mg/mL. L6 cells were treated with C/M for 0.5, 2 and 4 h as indicated. P values are from pairwise t-test with Holm correction and vs. C/M from untreated BMDMs (0 mg/mL). e) Western blots for other lectins in heart lysates. Complements main manuscript Fig. 5g,h. f) Western blots probing for sialic acid through MALII lectin binding in the liver from mice injected with ePL or PBS vehicle control.


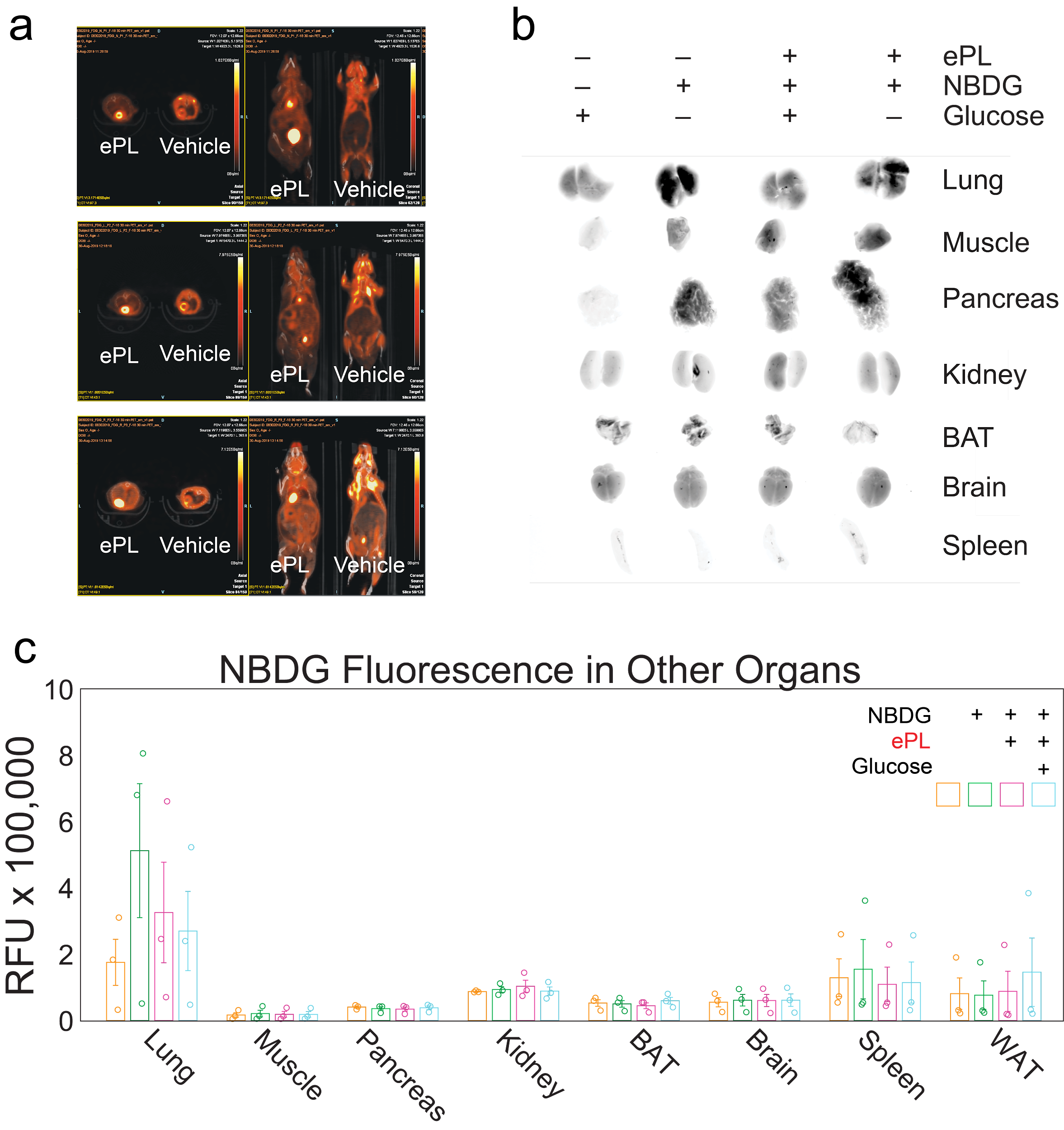


**Supplementary Fig. 5.** a) PET scan images used to quantify heart uptake in Fig. 6b of the main manuscript (n = 3 mice per group). b) Representative fluorescence images of whole organ imaging after NBDG injection. c) Quantification of NBDG uptake in various organs (n = 4 mice per group).

#

# Supplementary Methods

*Synthesis of virus-like nanoparticles (VLNPs)*

VLNPs were designed to have a carbohydrate coat and adeno-associated virus-like hydrodynamic diameter of ~25 nm. To this end, FluoSpheres latex polystyrene nanoparticles (Thermo Fisher, F8783) of 24 ± 3 nm in size labeled with dark red fluorophore (excitation/emission 660/680 nm) were coated with a lipid-conjugated maleimide to allow for orthogonal reaction with thioglucose. First, lipid-latex hybrids were synthesized as previously described by us.[^3^](https://paperpile.com/c/11UBca/8poaf) Briefly, 100 µg of dry DSPE-MAL (Nanosoft polymers, 10791), obtained from the evaporation of a 100 µL of 1 mg/mL solution in chloroform, was briefly bath-sonicated in 0.75 mL PBS buffer (0.2 M, pH 7.2), following which 0.1 mL of FluoSpheres (2% v/w) was added and the sonication continued for 10 min on ice. To the resulting lipid-latex hybrid, 0.15 mL of 10 mg/mL 1-thio-β-D-glucose sodium salt (Chem Impex, 27753) in PBS (0.2 M, pH 7.2) was added and the sonication continued for another 10 min on ice in the tube flushed with argon gas. The reaction mixture was allowed to warm to room temperature and then pulse-sonicated (1s, 5 min interval) in the water bath for 2 h under argon gas. The resulting fluorophore-labeled VLNPs were purified by ultracentrifugal precipitation (3 times, 100,000 g x 10 min) and dialyzed against 10 mM PBS (pH 7.2) with at least three buffer changes using 300 kDa dialysis membrane (Repligen, 131456). Agarose gel electrophoresis analysis (1% agarose) with subsequent staining using Pierce Glycoprotein Staining Kit (Thermo Fisher, 24562) confirmed the successful VLNP glycosylation. VLNP size was determined by DLS as described above.

*Quantitative real time PCR*

Extracted organs were processed by Macherey-Nagel ​​NucleoSpin Tissue kit (Takara Bio, 740952.250); AAV DNA was prepared with the same kit to produce a standard curve. Samples and standards were transferred to a 384-well plate and a Thermo Fisher Taqman MasterMix solution (4444557), forward and reverse primers for CMV sequence, and the CMV probe (CMV-Forward TTC CTA CTT GGC AGT ACA TCT ACG, CMV-Reverse GTC AAT GGG GTG GAG ACT TGG, CMV-Probe TGA GTC AAA CCG CTA TCC ACG CCC A) were added. Real-time quantitative PCR was performed on a Roche480 Lightcycler by the following protocol: hold at 50°C for 2 minutes, hold at 95°C for 20 seconds, then 40 cycles of 95°C denaturing for 3 seconds and 60°C annealing for 30 seconds.

*Immunoblot (Western blot) analysis*

Tissue samples were lysed in GoldBio Tissue Lysis Buffer (GoldBio, GB-181-100). Cultured cells were lysed using SDS loading buffer containing 2% sodium dodecyl sulfate (SDS), 100 mM dithiothreitol, 20% Ficoll 400, 0.1% bromophenol blue, and 60 mM Tris at pH 6.7. Total protein concentration was determined either by FTIR via a Millipore Sigma Direct Detect spectrometer or a BCA protein assay (Thermo Fisher, 23225). Samples were then loaded at a normalized protein concentration on Criterion TGX precast gels (Bio-Rad, 5671105) and run in an SDS running buffer for 20-25 minutes at 300V on a BioRad PowerPac HV system. Bio-Rad All Blue protein standard (Bio-Rad, 1610373) was run on each gel for molecular weight references. Proteins were transferred by electroblotting onto a PVDF membrane in the Bio-Rad TransBlot Turbo buffer on a BioRad TransBlot Turbo Transfer System. Membrane transfer was assessed by Ponceau staining (0.1% Ponceau in double distilled water with 0.5% acetic acid). Membrane was next blocked in 5% milk (when probing with lectins, milk was substituted by protein-free blocker, Thermo Fisher, 37585) in Tris-buffered saline containing 0.05% Tween 20 (TBST) for 1h. After washing with TBST, the membranes were probed with the following primary antibodies: anti-GFP (Thermo Fisher, A10262, at 1:5000 dilution), anti-phophoSTAT1 (Cell Signaling, 9167S, at 1:1000 dilution), anti-STAT1 (Cell Signaling, 14994S, at 1:1000 dilution), anti-phosphoPDH (Cell Signaling, 37115S, at 1:1000 dilution), anti-PDH (Cell Signaling, 2784S, at 1:5000 dilution), anti-paucimannose (Developmental Studies Hybridoma Bank, Laz6-189/Mannitou, at 1:75 dilution), anti-SLC35A1 (Proteintech, 16342-1-AP, at 1:1000 dilution), or biotin-conjugated lectins (*Maackia Amurensis Lectin I* (MAL I), B-1315-2; *Maackia Amurensis Lectin II* (MAL II), B-1265-1; *Ricinus Communis Agglutinin I*, B-1085-5; *Sambucus Nigra Lectin*, B-1305-2, all from Vector Laboratories and used at 1:2000 dilution). The probing was accomplished in 2.5% BSA or protein-free blocker in TBST overnight at 4°C with shaking. Next, the membrane was again washed with TBST before addition of secondary antibodies or streptavidin, conjugated with horseradish peroxidase (Thermo Fisher, A27036, 21130) at 1:50000 dilution in 2.5% BSA in TBST. This was incubated at room temperature for one hour with shaking, protected from light. The membrane was then washed with TBST. Detection solution was composed of luminol, 4-iodophenylboronic acid, and H_2_O_2_ in 100 mM Tris, pH 8.8, as previously described.[^4^](https://paperpile.com/c/11UBca/LAyO6) Detection solution was added to the membrane for 1-3 minutes before imaging on an Azure Biosystems C400 imager. When necessary, membranes were stained with Coomassie Brilliant Blue (CBB) to detect total protein content in the event of poor Ponceau image quality. The images were analyzed using Azure Spot software.

*Southern blotting and muscle analysis*

Skeletal muscle isolated from hindlimb and soleus/gastrocnemius was subjected to the DNA extraction as described above. The eGFP transcripts were then amplified using the following primers: eGFP-Forward CTG GTC GAG CTG GAC GGC GAC G, eGFP-Reverse CAC GAA CTC CAG CAG GAC CAT G. The amplification was accomplished using Platinum SuperFi II DNA Polymerase (Thermo Fisher, 12361010) according to the manufacturer’s instructions. Next, the Southern blotting was performed as previously described by us.[^5^](https://paperpile.com/c/11UBca/2l3jx) Briefly, the PCR product was resolved on 1% agarose gel, transferred to the positively-charged nylon membrane and hybridized with a specific digoxigenin-labeled probe (TGC ACG CTG CCG TCC TCG AT/3Dig_N) at 100 pM in ULTRAhyb Ultrasensitive Hybridization Buffer (Thermo Fisher, AM8670) at 42^o^C overnight. After stringency washes with 2 and 0.2% SSC (saline-sodium citrate buffer, Millipore-Sigma SRE0068) at room temperature and 42^o^C respectively (15 min, 3 times each), the digoxigenin label was detected using Roche DIG DNA Labeling and Detection Kit (Millipore-Sigma, 11093657910) according to the manufacturer’s instructions.

*Liver cells isolation*

Primary mouse liver cells were isolated as described previously.[^6^](https://paperpile.com/c/11UBca/30LN6) Briefly, mice under anesthesia with 5% isoflurane were carefully perfused with Hanks' Balanced Salt Solution (HBSS, Thermo Fisher, 14175095) through portal vein until the liver turned to pale yellow before switching to liver digestion buffer, which was prepared by dissolving 1 mg/mL of Collagenase Type IV (Worthington Biochemical Corporation, LS004188) in HBSS supplemented with 1 mM calcium chloride and kept warm at 37℃ in a water bath. Each liver was perfused with the liver digestion buffer for at least 3 min and then smashed through a 100 μm cell strainer. The cell suspension was centrifuged at 50 g for 2 min and washed with 2% FBS in Dulbecco's Modified Eagle Medium (DMEM) twice.

*Flow cytometry*

Liver cells were isolated as described in the previous section and resuspended in 0.5% BSA in HBSS at 10^6^ cells/mL. The cells were then stained for 1 hour on ice with Live/Dead (Thermo Fisher, L34957), anti-CD3 (Biolegend, 100217), anti-CD4 (Biolegend, 100456), anti-CD11b (Biolegend, 101210), anti-CD25 (Biolegend, 102005), anti-CD68 (Biolegend, 137021), anti-CD146 (Biolegend, 134703), anti-CD206 (Biolegend, 141727), anti-F4/80 (Biolegend, 123118), and anti-Ly6G (Thermo Fisher, 25-5931-82). After incubation, the cells were washed with 0.5% BSA in HBSS and pelleted by centrifugation at 400 g for 5 min. The pellets were resuspended in 2% paraformaldehyde in PBS for flow cytometry analysis.

*Luciferase assays*

Promega luciferase assay system was utilized in testing with AAV-fLuc, encoding for firefly luciferase. Samples (cells or tissue homogenates) were lysed in cell culture lysis buffer (Promega, E153A). Despite the nomenclature, this buffer is appropriate for tissue homogenate as well as cells (confirmed both in Promega’s system manual and via communication with technical representatives). Cells were scraped in a lysis buffer and observed under a microscope to ensure they had been fully lysed before proceeding. Tissue homogenates were lysed in 200-300 μL cell culture lysis buffer by rotating at 4°C for at least 30 minutes and then undergoing three freeze-thaw cycles by alternating between liquid nitrogen and a 37°C bead bath. Samples were centrifuged for 2 min at 14800 rpm and supernatant was collected. In an opaque white-walled 96-well plate, 20 μL of sample (cell or tissue lysate) was added. A Molecular Devices SpectraMax L plate reader was used to dispense 100 μL of Promega luciferase assay reagent (Promega, E1483) into each well and measure the resulting luminescence using a 10 s integration time. Luminescence was normalized against total protein content, measured by BCA assay (Thermo Fisher, 23225).

*Nanostring analysis*

Bone marrow derived macrophages (BMDMs) were treated with ePL at 1 mg/mL or PBS vehicle control for 30 min or 2 h, followed by a wash with cold PBS and addition of an RNA lysis buffer from Macherey-Nagel NucleoSpin RNA Plus kit (Takara Bio, 740984.50). The RNA was then isolated according to the manufacturer’s instructions. The RNA was subjected to the standard Nanostring assay using nCounter Inflammation Panel (Nanostring, XT-CSO-MIM1-12). The analysis was performed at the Michigan State University Genomics Core. The counts were analyzed using nSolver analysis software with the counts normalized to a panel of housekeeping genes (*Ppia*, *Hprt*, *RPL19*, *SDHA*, *TUBB*, *ABCF1*, *GUSB*, *ALAS1*).

*Immunofluorescence microscopy*

Paraffin-embedded tissue was cut to a thickness of 8 µm onto glass slides followed by the standard dewaxing/hydration technique through a series of graded xylene-ethanol-water washes. The slides were blocked using Fish Serum Blocking Buffer (Thermo Fisher, 37527) containing 0.2% Triton X-100 and incubated with either anti-cardiac troponin T monoclonal antibody (Developmental Studies Hybridoma Bank, CT3) at a 1:100 dilution or with anti-GFP polyclonal antibody (Thermo Fisher, A6455) at a 1:200 dilution. The incubation proceeded overnight in the blocking buffer at 4^o^C, following which the slides were washed with PBS three times and incubated with either goat anti-mouse IgG2a Alexa Fluor 488, or with goat anti-rabbit Alexa Fluor 488 secondary antibodies (Thermo Fisher, A-21131 and A-11008) both at a 1:250 dilution for 1 h at room temperature. After three washes with PBS, the slides were mounted using Vectashield antifade mounting medium with DAPI (Vector Laboratories, H-1200-10) and visualized on Keyence BZ-X 700 fluorescence microscope.

L6-GLUT4myc rat myotubes were cultured in 8-well chambered slides (Ibidi, 80826), treated with cell culture supernatants or 100 nM insulin (Alfa Aesar, J61321-MD) for 1 min as described in the main text and below. After a quick wash with cold PBS, the cells were fixed in 4% paraformaldehyde for 10 min at room temperature. After three washes with PBS, the cells were blocked as described above for tissue slides and incubated with anti-cMyc polyclonal antibody (Millipore-Sigma, C3956) at a 1:200 dilution for 1 h at room temperature. After three washes with PBS, the cells were incubated with goat anti-rabbit Alexa Fluor 488 secondary antibody as above, and stained with Hoechst 33342 (Thermo Fisher, H1399) at 1 µg/mL for 10 min at room temperature. The washed slides were then visualized using Keyence BZ-X 700 fluorescence microscope.

*Image analysis*

Whole organ fluorescence was analyzed using ImageJ/Fiji, an open-source image analysis software. In brief, the images were first color inverted and the ROIs were created by tracing each organ. The integrated intensity of fluorescence per ROI area was then determined using the “Analysis” function in ImageJ. After autofluorescence subtraction, the values of each organ were normalized to the value of liver from the same animal. The analysis of eGFP expression in the heart and liver was performed using Keyence BZ-X analyzer and a Hybrid Cell Count plugin. First, the area of analysis was identified using the “Same Intensity” function and then the “Cell Separation” function was used to outline individual eGFP+ cells. The number of eGFP+ cells was next counted in each slide using a premade macro function and a “Macro cell count” module. Ten slides at different histopathological locations (30 µm difference between slides) were analyzed per mouse/condition.

*Intravital microscopy (IVM)*

C57BL/6 male mice (8 weeks of age) were anesthetized with ketamine (80 mg/kg) and xylazine (7 mg/kg) cocktail and the hair was removed from their left ear using a Nair lotion applied to a cotton swab. After lotion removal with warm PBS, a tail vein catheter was inserted. The animal was placed onto a heated stage (37^o^C) with its ear immobilized by two-sided taping to a Tokai Hit Thermoplate glass heater (TPi-SQFTX) adjusted to 37^o^C. This allowed for the animal to maintain whole body temperature and created a window of observation where the ear was positioned. The animal’s body temperature was monitored continuously *via* an electronic rectal thermometer. The heater was affixed into a Keyence BZ-X 700 fluorescence microscope stage. The imaging was performed using a 20x objective and a time-lapse video module in the BZ-X Analyzer. First, blood vessels in the ear were located by illuminating the ear with a white light. After adjusting xy-position and focus, the imaging was continued under the green fluorescence channel (GFP cube, Chroma Technology, 39002) at 640x480 pixel resolution and 30 frames per second. After localization and acquisition of the pre injection scan, each mouse was subjected to an i.v. injection of ePL immediately followed by FluoSpheres latex polystyrene nanoparticles (Thermo Fisher, F8787) via catheter. ePL was administered at 30 mg/kg and FluoSpheres were injected at 1 mg/kg in a PBS solution. Imaging was performed continuously for 1 h and terminated early if a subject became conscious. The videos were analyzed using BZ-X Analyzer.

*Blood clearance analysis and pharmacokinetics of AAVs*

A C57BL/6J mouse was injected with 2.25 vg/kg AAV9-CMV-GFP intravenously (retro-orbitally) and a 1 μL blood sample was taken from its tail via a 1 mm tail snip at 1, 2, 6, 20 and 28 hours. Blood was processed with the RedExtract-N-Amp Blood PCR kit (Millipore-Sigma, XNABR-1KT). Samples were lysed in the provided lysis solution at room temperature and neutralized overnight at 4°C in the provided buffer before being analyzed directly by qPCR, as described above. Whole blood spiked with known concentrations of AAV9 was used to generate the standard curve. dCT values obtained from the qPCR were fitted against viral vector genome titers (VG) in the standard curve using a five parametric curve fitting to calculate VG titers per µL of blood.

*Lec2 neutralizing antibody assays*

Lec2 cells were cultured in α-MEM (Thermo Fisher, 12571063) with 1% penicillin/streptomycin and 10% fetal bovine serum (FBS). Transduction with AAV9-fLuc was accomplished in a 96-well clear-bottomed, white opaque-walled plate. The experiments were performed in the presence of serum from mice that had received either AAV9, AAV9 and ePL, or left untreated. Serum was collected from mice 30 days after the injection and kept frozen at -80°C before use. Additionally, in one group, the cells received ePL along with AAV9-serum. Serum was dosed in cells at 1:10 dilution, and ePL was dosed at 1:100 (0.1 mg/mL). AAV9-fLuc was added to cells at an MOI of 50000 vector genomes per cell. After 24 h, transduction media was removed and the cells were lysed and analyzed by luminescence reporter assay, as described above.

*Isolation of primary cardiomyocytes and calcium influx imaging*

Primary cardiomyocytes were isolated as described previously from male C57BL/6 mice of 6-8 weeks of age.[^7,8^](https://paperpile.com/c/11UBca/jBvC6+4rmcA) Briefly, after euthanasia, the hearts were removed and enzymatically digested in Krebs-Henseleit buffer (118 mM NaCl, 4.7 mM KCl, 1.2 mM MgSO_4_, 1.25 mM CaCl_2_, 1.2 mM KH_2_PO_4_, 25 mM NaHCO_3_, 11 mM glucose) supplemented with 68 mM blebbistatin (Millipore-Sigma, 203391) and 268 IU/mL type II collagenase (Worthington Biochemical Corporation, LS004177). After enzymatic digestion and wash, the cells were plated and cultured on laminin coated coverslips (Thermo Fisher, 23017015) at 20,000 myocytes per coverslip in M199 media supplemented with 1% penicillin/streptomycin and 5% fetal bovine serum at 37ᵒC, 5% CO_2_, 21% O_2_ for 2 h to allow for complete cell attachment.

Myocyte contractility was recorded in real time on the IonOptix recording system equipped with a high-speed CCD camera. The cells were paced at 0.5, 1, 2, 3, and 4 Hz and only myocytes with a resting sarcomere length more than 1.75 μm were included in the following experiments. Next, the cells were placed in a chamber maintained at a 37º ± 1ᵒC and perfused with Tyrode’s Solution (Millipore-Sigma, T1788). The cells were loaded with Fluo-4 AM at 10 µM (Thermo Fisher, F14201) containing 0.2% Pluronic F-127 for 40 minutes at room temperature followed by a 40 min wash time. Next, the cells were treated with different cell conditioned mediums (C/Ms) obtained from BMDMs treated as described above with ePL, untreated or incubated with 25 mM sodium lactate for 24 h. Direct treatment of cardiomyocytes at 1 mg/mL of ePL or 25 mM sodium lactate was also carried out. All treatments proceeded at 37ᵒC for 1 h. Base M199 medium served as a baseline control. Calcium was introduced in two steps (1 mM and 1.8 mM) in Krebs-Henseleit buffer supplemented with 25 µM blebbistatin. Myocyte fluorescence (emission at 510 nm) was recorded using a photomultiplier tube when excited with 340 nm (calcium independent measure) and 380 nm (calcium dependent measure) excitation wavelengths. Ionwizard software (IonOptix, LLC) was used to analyze the results. Diastolic calcium, peak amplitudes, shortening velocity, relaxation times, and reuptake times were calculated for each pacing frequency.

*L6-GLUT4myc plate assay*

Rat myoblast L6 cells engineered to carry Myc-tagged GLUT4 were cultured and differentiated as suggested by their commercial distributor Kerafast. Next, differentiated myotubes were plated in 48-well plates at 250,000 cells per well in α-MEM (Thermo Fisher, 12571063) followed by the attachment period of 12 h. The cells were then treated with 0, 1 and 2 mg/mL ePL or incubated at 1 mg/mL for 30 min, 1 h, and 4 h. In some experiments, the cells were treated with insulin at 100 nM (Alfa Aesar, J61321-MD) for 1 min before the end of the incubation period. The plates were then washed with cold PBS and fixed in 4% paraformaldehyde at room temperature for 10 min. After three washes with PBS, the cells were blocked in 5% nonfat dry milk in PBS-T (PBS with 0.05% Tween 20) and incubated with rabbit anti-cMyc polyclonal antibody (Millipore-Sigma, C3956) at a 1:200 dilution in PBS-T for 1 h at room temperature. After three washes with PBS-T, the cells were incubated with goat anti-rabbit HRP-conjugated superclonal recombinant secondary antibody (Thermo Fisher, A27036) at 1:1000 dilution in 5% nonfat dry milk in PBS-T for 1 h at room temperature. Next, the plates were washed with PBS-T three times, the wash solution was completely removed by suction, and 150 µL of o-phenylenediamine dihydrochloride (obtained from OPD tablets, Thermo Fisher 34006) solution in Stable Peroxide Substrate Buffer (Thermo Fisher, 34062) was next added and incubated for 15 min. To stop the enzymatic reaction, 50 µL of 2.5 M sulfuric acid was added to each well and the optical absorbance was measured at 490 nm using a plate reader.

*Positron emission tomography*

The imaging was performed at The Case Center for Imaging Research (CCIR). C57BL/6 mice of 8 weeks of age (n=3 per group) were fasted before the experiments for 6 h. Mice were injected with ePL at 30 mg/kg or vehicle PBS followed by intraperitoneal injection with insulin (Humulin R, Medline Industries, 0002-8215-17) at 0.75 mU/Kg, and 15 min later anesthetized with ketamine (80 mg/kg) and xylazine (7 mg/kg) cocktail. This was immediately followed by an i.v. injection of 2-deoxy-2-[^18^F]fluoro-D-glucose (FDG) at 300 µCi. Next, a whole-body micro-computed tomography (CT) scout view was acquired to confirm animal positioning and to determine field of view. Immediately after, the CT acquisition scan was performed to allow for attenuation correction. Thirty minutes after FDG injection, positron emission tomography (uPET/CT, Siemens Medical Solutions) and a whole-body CT (uPET/CT, Inveon, Siemens Medical Solutions) imaging were performed. A CT scan was acquired at 80 kVp energy, 900 μA current, and 2.3 micrometer voxel size. Quantitative image analysis of the FDG uptake in various organs was performed using Carimas II Research Workplace software. First, regions of interest (ROIs) were identified from the reconstructed uPET frames followed by the quantification of the standard uptake value (SUV) in an ROI. Based on the PET and CT co-registered images the following sites/organs were identified and assigned specific ROIs: brain, heart, liver, muscle, white adipose tissue (WAT), and brown adipose tissue (BAT). The uptake was calculated using the mean SUV across three animals per group. PET imaging was performed under CCIR protocol (2015-0059), which allows for handling radioactive materials in live animals.

# Uncropped Western Blotting Images

**
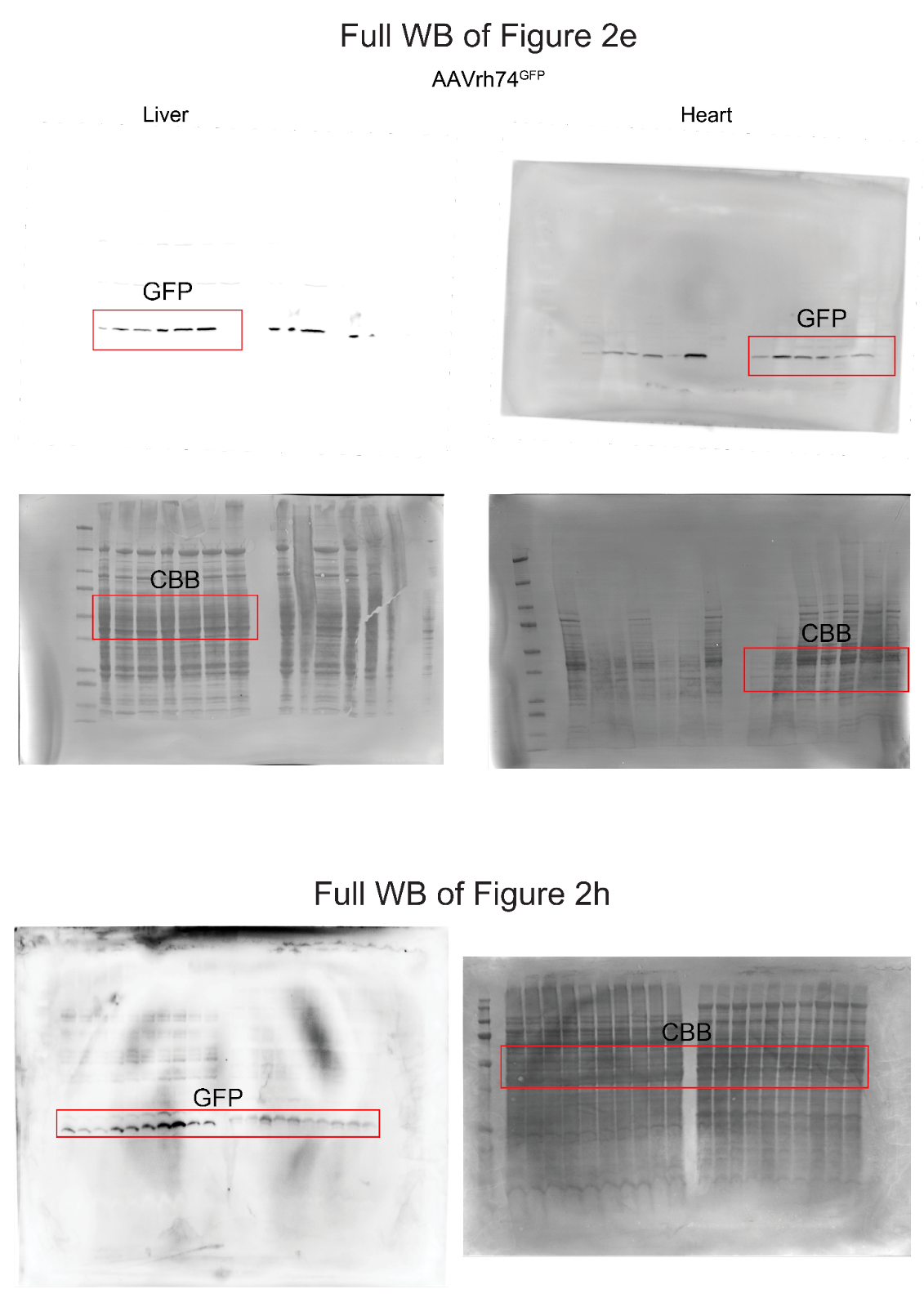
**

**
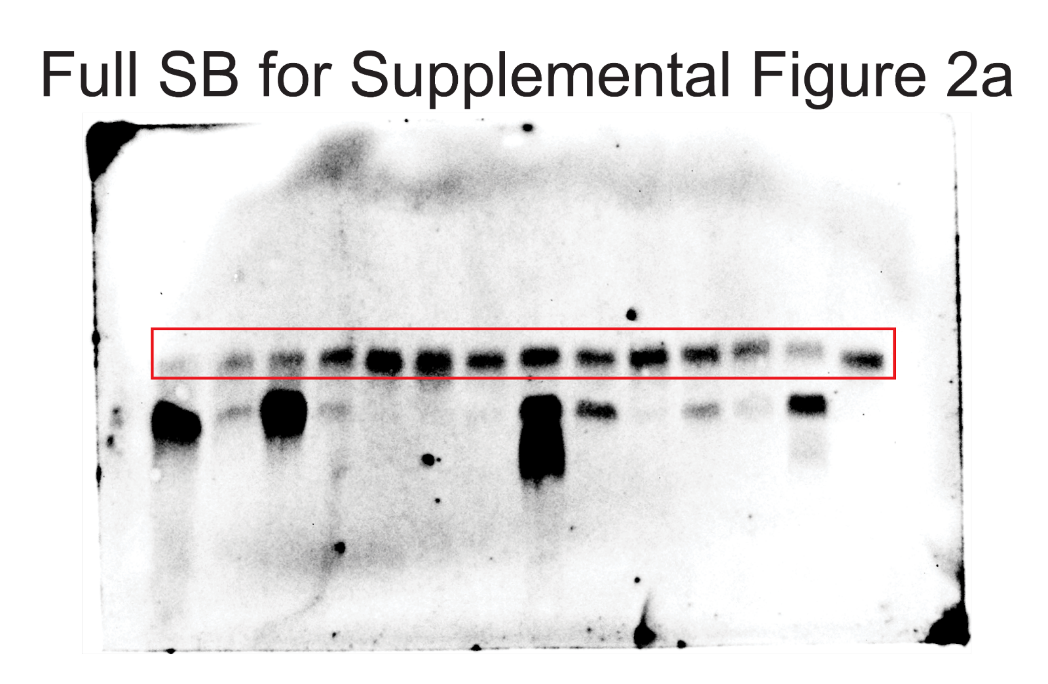
**

#
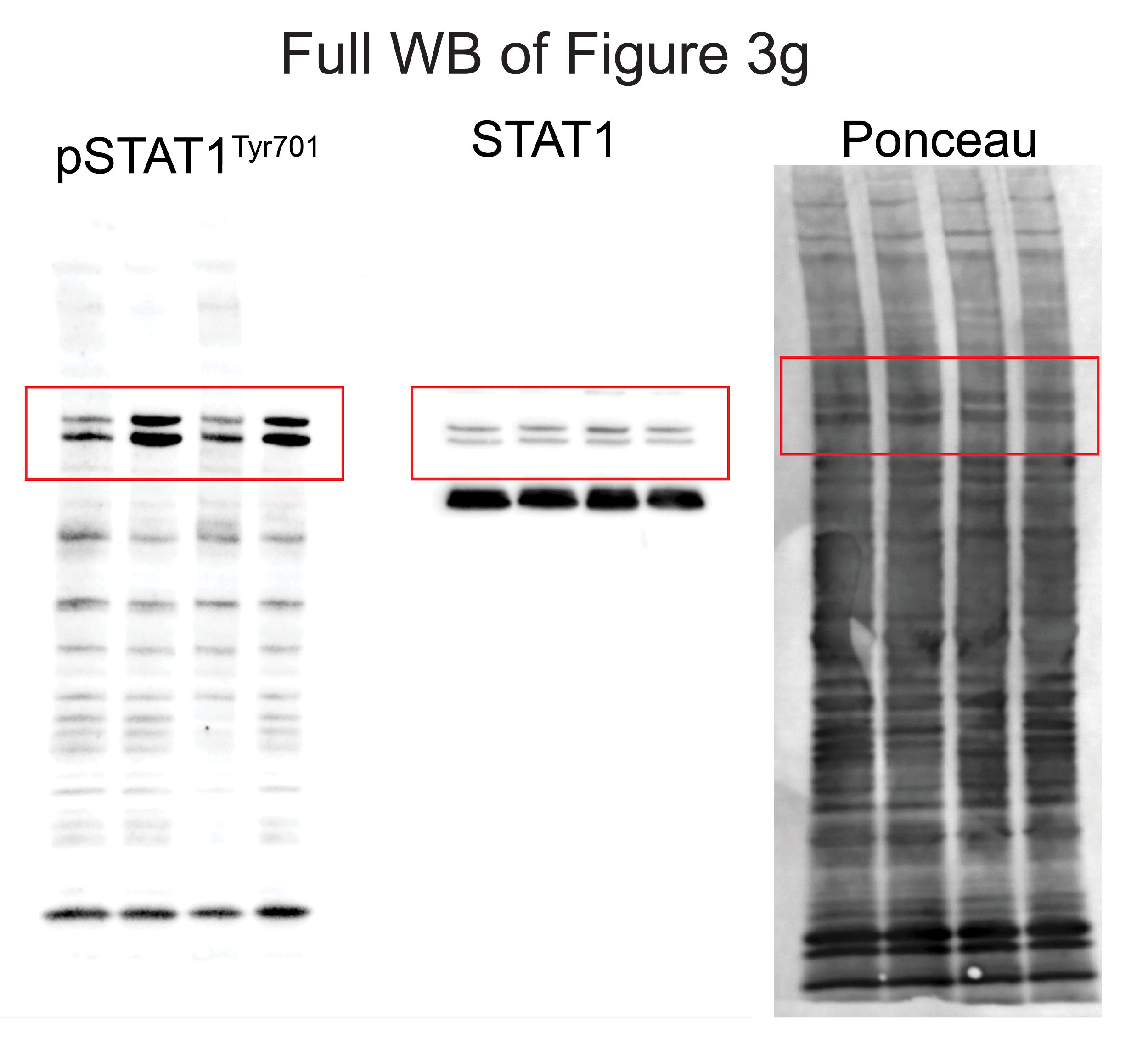

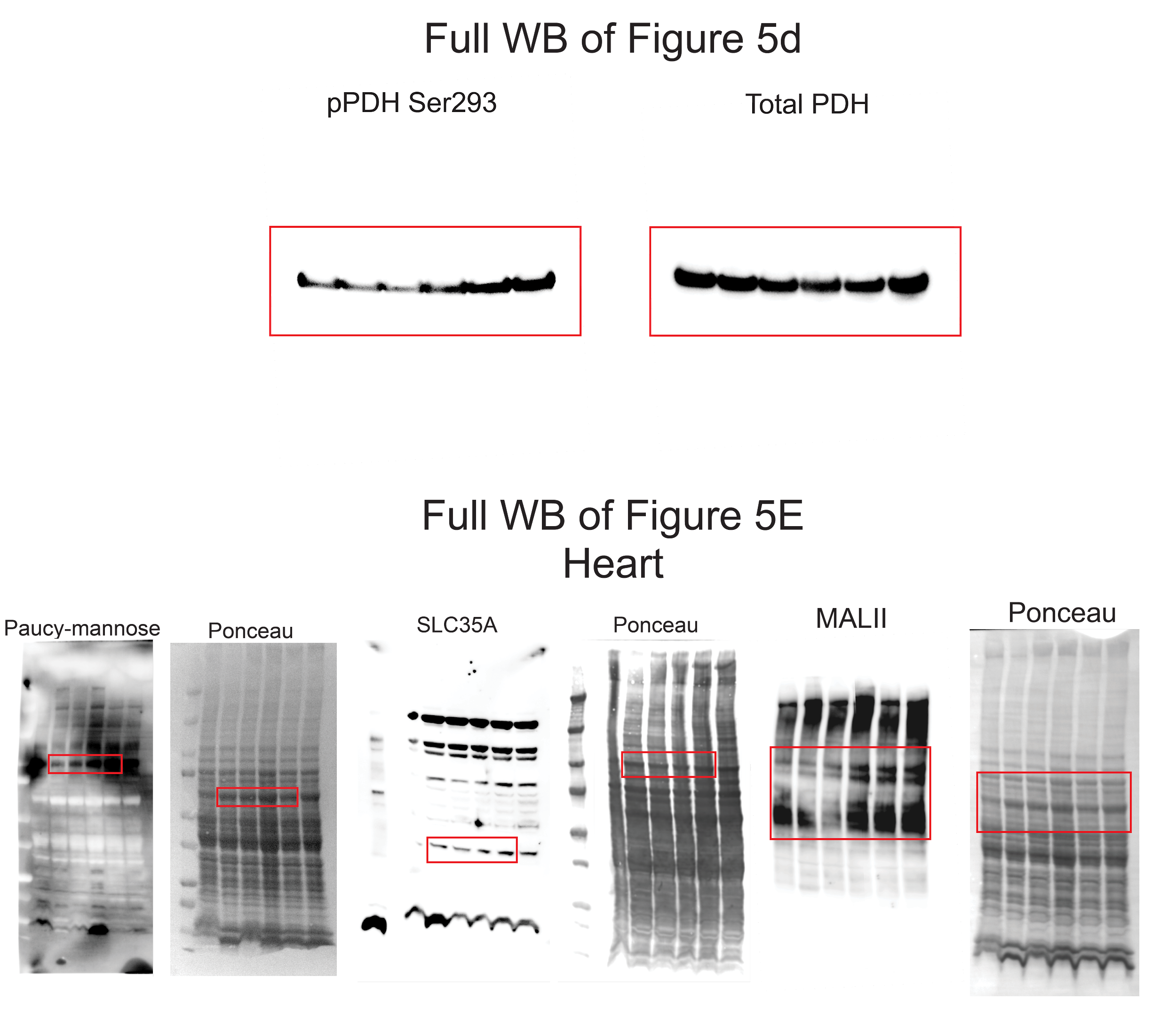


#

# References

1. [Sharma, S. & Haldar, C. Comparative effect of melatonin and vitamin E on phenylhydrazine-induced toxicity in the spleen ofFunambulus pennanti. *Environmental Toxicology* vol. 24 1–9 Preprint at https://doi.org/](http://paperpile.com/b/11UBca/a8dJo)[10.1002/tox.20383](http://dx.doi.org/10.1002/tox.20383) [(2009).](http://paperpile.com/b/11UBca/a8dJo)

2. [Pandey, K., Meena, A. K., Jain, A. & Singh, R. K. Molecular mechanism of phenylhydrazine induced haematotoxicity: A review. *Ame J Phytomed Clin Therapeut* **2**, 390–394 (2014).](http://paperpile.com/b/11UBca/kAYx8)

3. [Bagalkot, V. *et al.* Hybrid nanoparticles improve targeting to inflammatory macrophages through phagocytic signals. *J. Control. Release* **217**, 243–255 (2015).](http://paperpile.com/b/11UBca/8poaf)

4. [Mog, B. *et al.* Nano-Antagonist Alleviates Inflammation and Allows for MRI of Atherosclerosis. *Nanotheranostics* **3**, 342–355 (2019).](http://paperpile.com/b/11UBca/LAyO6)

5. [Maiseyeu, A. *et al.* Plaque-targeted, proteolysis-resistant, activatable and MRI-visible nano-GLP-1 receptor agonist targets smooth muscle cell differentiation in atherosclerosis. *Theranostics* **12**, 2741–2757 (2022).](http://paperpile.com/b/11UBca/2l3jx)

6. [Cabral, F. *et al.* Purification of Hepatocytes and Sinusoidal Endothelial Cells from Mouse Liver Perfusion. *J. Vis. Exp.* (2018) doi:](http://paperpile.com/b/11UBca/30LN6)[10.3791/56993](http://dx.doi.org/10.3791/56993)[.](http://paperpile.com/b/11UBca/30LN6)

7. [Han, S. *et al.* Optical Imaging of Isolated Murine Ventricular Myocytes. *Journal of Visualized Experiments* Preprint at https://doi.org/](http://paperpile.com/b/11UBca/jBvC6)[10.3791/60196](http://dx.doi.org/10.3791/60196) [(2020).](http://paperpile.com/b/11UBca/jBvC6)

8. [Klos, M., Morgenstern, S., Hicks, K., Suresh, S. & Devaney, E. J. The effects of the ketone body β-hydroxybutyrate on isolated rat ventricular myocyte excitation-contraction coupling. *Arch. Biochem. Biophys.* **662**, 143–150 (2019).](http://paperpile.com/b/11UBca/4rmcA)
